# Origin and timing of spilitic alterations in volcanic rocks from Głuszyca Górna in the Intra-Sudetic Basin, Poland

**DOI:** 10.1038/s41598-022-15644-2

**Published:** 2022-07-11

**Authors:** Tomasz Powolny, Magdalena Dumańska-Słowik, Aneta A. Anczkiewicz, Magdalena Sikorska-Jaworowska

**Affiliations:** 1 Faculty of Geology, Geophysics, and Environmental Protection, AGH – University of Science and Technology, 30 Mickiewicza Av., 30-059 Kraków, Poland; 2 Institute of Geological Sciences PAS, 1 Senacka St, 31-002 Kraków, Poland; 3 Polish Geological Institute - National Research Institute, 4 Rakowiecka St, 00-975 Warsaw, Poland

**Keywords:** Solid Earth sciences, Core processes, Geochemistry, Geodynamics, Geology, Mineralogy, Petrology, Volcanology

## Abstract

The formation of spilitic assemblages (i.e. chlorite and albite) has been ubiquitously involved during the evolution of continental early-Permian volcanics from the Intra-Sudetic Basin (ISB). Based on the investigation of laccolith-type and variably-altered trachyandesite exposure in the vicinity of Głuszyca Górna (Lower Silesia, Poland), we have demonstrated that apatite fission-track dating (AFT), coupled with chlorite geothermometry, can be successfully applied to denote the timing of low-temperature alterations within volcanic rocks. The primary magmatic assemblages of the trachyandesites (i.e. augite and andesine-labradorite) have been affected by chloritization and alblitization respectively, followed by the formation of secondary titanite, celadonite, and calcite. The chlorite species have crystallized in the range of 106–170 °C, that exceeds Apatite Partial Annealing Zone (70–110 °C). The secondary, nearly pure albite (Ab ~ 99 mol.%) with weak to dark-brown cathodoluminescence replaces primary plagioclase (~ An_37–50_Ab_47–58_Or_2–4_) along the cleavage and/or twinning planes during Al^3+^–conservative reaction. The accessory apatite is marked by swallow-tail terminations indicative of rapid cooling formation conditions. It shows homogenous chemical composition, high F^−^ content, and pink to yellow (REE^3+^ and Mn^2+^-activated, respectively) cathodoluminescence. Based on the AFT dating, the development of spilitic alterations within the early-Permian (ca 290 Ma) laccolith from Głuszyca could not only span the range of 182–161 Ma (Middle Jurassic), but also occurred prior to large-scale geological events in the ISB, such as burial under late-Mesozoic sediments, as well as tectonic inversion and exhumation. Whole-rock geochemistry of trachyandesites altered to various extent, indicates that original trace elements concentrations, except for i.e. Sr, Cs, and Ba, could be preserved during low-temperature alteration (spilitization). Meanwhile, geochemical fingerprint of the volcanics (i.e. humped-shaped mantle normalized trace element diagrams and positive Zr–Hf anomaly) points to the crustal contamination during magma evolution, combined with the mantle metasomatism in the source via subduction-derived components (i.e. fluids), as shown by i.e. low Nb/Th and Nb/LREE ratios.

## Introduction

Albitization (Na-metasomatism) is a prevalent hydrothermal-metasomatic process in the upper crust of the Earth, which results in the breakdown of pre-existing Ca-rich plagioclases and/or alkali-feldspars into secondary, nearly pure albite^1,2^. It has been reported from various geological settings all around the world (i.e.^3^), and usually acts via either interface-coupled dissolution-reprecipitation mechanism or solid-state diffusion or both of them—e.g.^4,5^. Albitization affects both igneous (chiefly granites and gabbroic rocks; see e.g.^6^) and sedimentary rocks such as sandstones^7^. This process is often spatially coupled with other alterations including scapolitization and silicification (^8^ and ^9^, respectively), but may also accompany ore-forming processes due to the remobilization of particular metals such as Cu and Au—see e.g.^10^. The temperature of albitization has been estimated using e.g. two-feldspar geothermometry^9^, oxygen isotope data^11^, and Raman-based carbonaceous material thermometry^12^. According to these studies, the formation of secondary albite may be regarded as either low (~ 70 °C) or up to medium-temperature (up to ca. 350 °C) process depending on the physicochemical properties of alternating fluids.

The formation of secondary albite in basaltic lavas, followed by the development of associated low-temperature (secondary) mineral assemblages such as chlorite, calcite, actinolite, prehnite, or epidote, commonly refers to so-called spilitization^13^. This process has been first described by Brongniart^14^ and leads to the formation of spilites (also known as spilitic rocks). The exact nature of spilites (so-called spilite problem^13,15^) is not entirely understood owing to the origin of albite-chlorite assemblage and corresponding source of Na^+^. Overall, spilitization has been reported from basic to intermediate rocks where it may originate from: (1) water–rock interactions during e.g. submarine eruptions or intrusions of magma into water-rich sediments, which are both capable of providing necessary amounts of Na^+^ for albitization, (2) auto-hydrothermal (late-magmatic or post-magmatic) alterations related to the interactions between early-formed mineral phases with coexisting residual (deuteric) fluids, and (3) low-grade metamorphic reactions during the burial stage^13^. Nevertheless, the primary nature of spilites—linked to either formation of the specific “hydrous”, Na-CO_2_-rich magma or mixing magma with hot brines—has been also proposed. However, the latter theory was nearly abandoned these days. Spilitization plays an important role in the recycling of nitrogen within the crust^16^ and may induce the development of carbonatization in the presence of dissolved CO_2_^17^. Furthermore, spilitization commonly obliterates original microtextural and mineralogical features of the rocks, but is also capable of overprinting their original geochemical signature, including main and trace element abundances (e.g. REE and LILE; e.g.^18^) and isotopic ratios (i.e. Sr and Nd; see:^19^). Hence, the isotopic homogenization during spilitic alterations has been thus used to constrain the timing of spilitization^19^.

Albitization (including spilitization) and K-metasomatism are conspicuously presented among early-Permian volcanogenic formations in Central Europe (see i.e.^20^). The spilitic assemblages, represented by albite, chlorite, riebeckite, and/or *uralite* have been particularly reported from upper parts of basaltic shallow-level magmatic bodies that cover a wide area of the Intra-Sudetic Basin (ISB) in Lower Silesia Poland^21^. In general, it has been accepted that albitization (spilitization) of early-Permian volcanics from the Intra-Sudetic Basin has resulted from late- to post-volcanic alkaline emanations that affected not only volcanic rocks, but also adjacent sediments^22^. Additionally, the formation of spilitic rocks in the ISB was spatially and genetically linked to the development of agate mineralization, as shown by Powolny et al.^23^.

In this paper, we have aimed at the reconstruction of mineral-replacement reactions during low-temperature alteration (spilitization) of continental volcanic rocks from the Intra-Sudetic Basin based on variably altered samples collected from laccolith-type exposure in the vicinity of Głuszyca Górna (Lower Silesia, Poland). Particularly, we show that combination of chlorite thermometry and apatite fission-track dating (AFT) can be successfully applied to establish the timing of low-temperature hydrothermal fluid flow responsible for the formation of spilitic assemblages in volcanic rocks. Finally, the whole rock major and trace element data provided a reconstruction model of magma evolution and the effects of secondary alterations on the possible redistribution of trace elements, such as REE and HFSE.

## Geological setting and field observations

The study area is situated within the Intra-Sudetic Basin and falls into NE flank of the Bohemian Massif, which in turn represents a large eastern exposure of European Variscan Belt in Central Europe. The ISB is filled with volcanogenic-sedimentary succession and exhibits a fault-bounded synclinorial structure of ca. 70 km long and 35 km wide that extends into NW–SE direction (e.g.^20,24^). It originally appeared as an intramontane depression confined by tectonically active margins^25^. The incipient stages of the ISB evolution began during Mid-Visean^26^ and were strictly related to the regression of the sea that appeared in SW Poland during the Devonian period. The Intra-Sudetic Basin is mantled by crystalline basement units of Variscan age and Late Palaeozoic sedimentary basins. According to Aramowicz et al.^27^, Sobczyk et al.^28^, and Botor et al.^29^, both volcanogenic and sedimentary rocks of the Bohemian Massif (including the Intra-Sudetic Basin) show quite complex thermo-tectonic histories imprinted by the sediment burial connected with transgression during the Cenomanian, Europe-Africa plate convergence followed by tectonic inversion and reactivation of faults and thrusts, as well as post-magmatic hydrothermal activity^30^.

**Figure 1:**
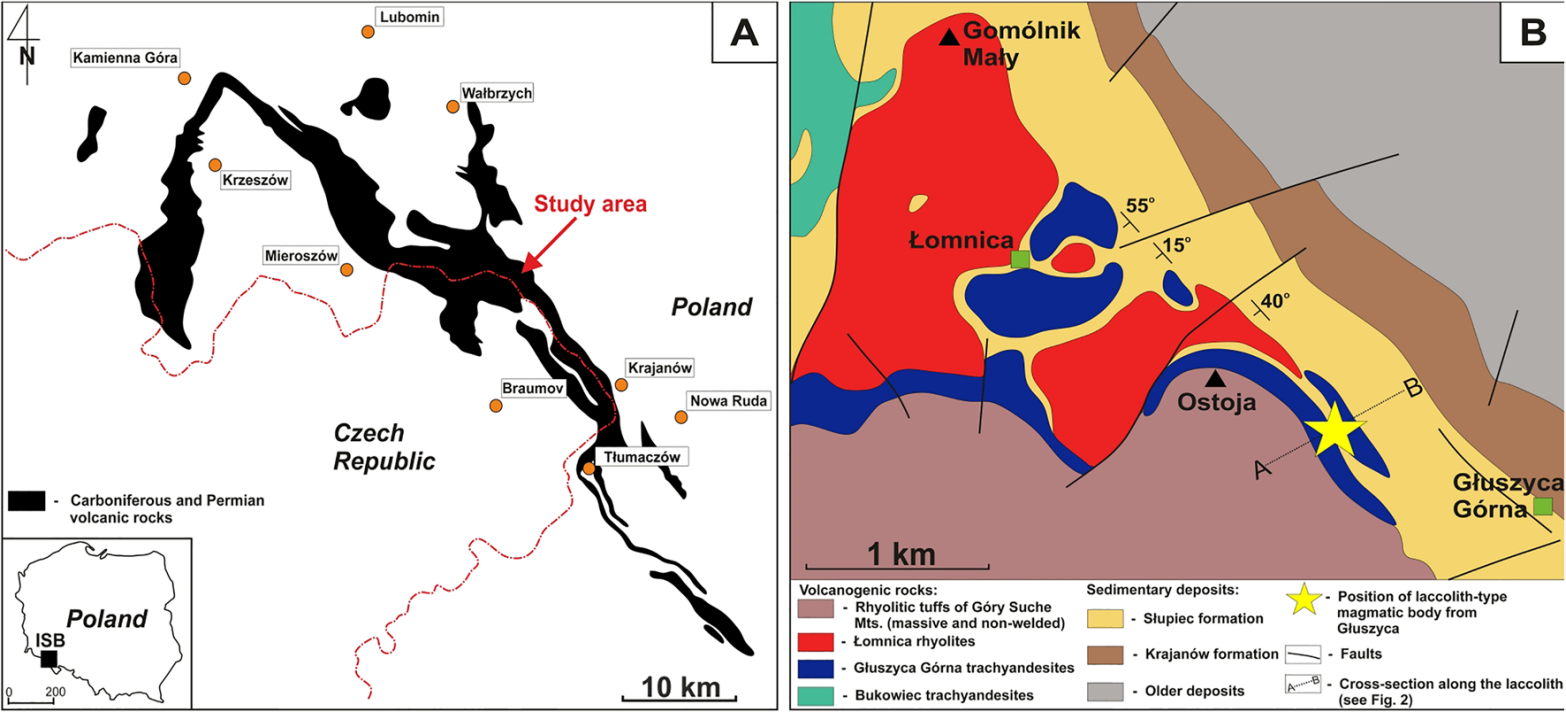
(**A**) Distribution of Carboniferous and Permian volcanic rocks within the Intra-Sudetic Basin (ISB)—modified after various sources, i.e.^24^. Note the inset map in the lower left show the position of the Basin within Poland area; (**B**) Geological sketch showing main lithologies found in the close vicinity of trachyandesite laccolith-type body exposed in the vicinity of Głuszyca village (after:^33^, modified). The map was generated using CorelDrawX6 software.

**Figure 2:**
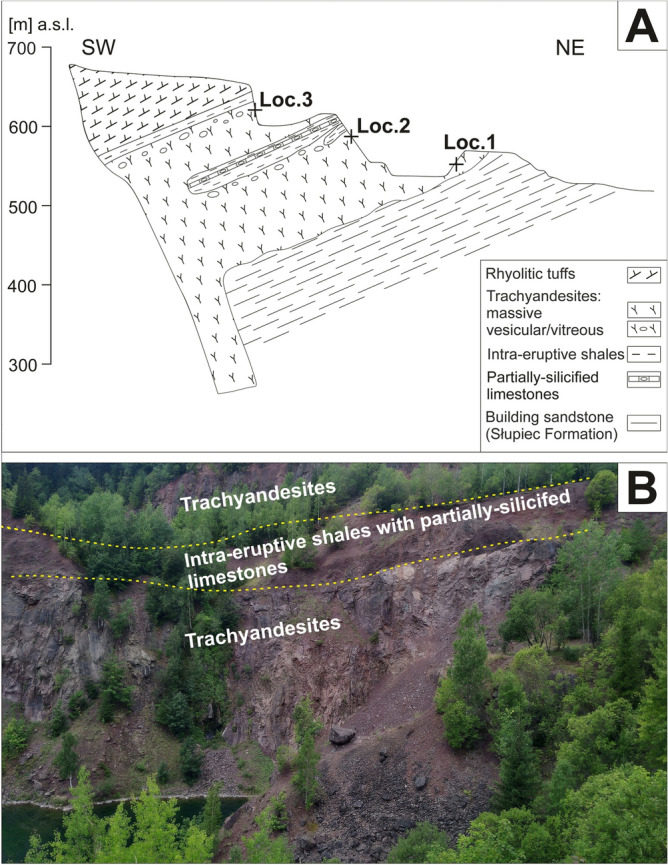
(**A**) Geological cross-section along A–B line (see Fig. 1B) of the subvolcanic laccolith-type magmatic body exposed abandoned Głuszyca quarry (after ^31^; modified via own field observations); Note the overall 3 sampling areas: Loc. 1 (lower parts of the laccolith), Loc. 2 (middle parts at the contact zone with adjacent intra-eruptive sediments), and Loc. 3 (top portions of the laccolith). (**B**) Field photo showing the contact between trachyandesites and intra-eruptive deposits (shales with partially-silicified limestones). The cross-section was generated using CorelDrawX6 software.

Głuszyca trachyandesite exposure of early-Permian age is located *ca.* 20 km SE from Wałbrzych and 15 km NW from Nowa Ruda (Lower Silesia, Poland)—Fig. 1A–B. It comprises a laccolith-type magmatic body of *ca.* 200 m in depth and 300 m in length^31,32^ that was actively exploited in the past. According to Kozłowski^31^ and Awdankiewicz^32^, the volcanics from the study area belong to the so-called second volcanic cycle of the Middle Rotliegendes and mildly-alkaline suite of the Rybnica Leśna Volcanic Association, respectively. The magmatic body reveals a well-developed, horizontal-oriented system of joint planes^31^. It lies over sandstones with interlocations of shales, but appears covered by acidic igneous rocks (i.e. rhyolitic tuffs)—Fig. 2A. The middle areas of the exposure reveal the presence of shales interbedded with carbonaceous limestones (the latter has been partially affected by post-magmatic silification)—see Fig. 2B.

The emplacement of volcanics from the Głuszyca exposure occurred during the climax of Variscan Orogeny and hence reflects the peak of magmatic activity developed within the ISB^31^. This magmatic activity has developed in subsidence-related areas such as ISB and resulted in the formation of alternating basic (i.e. trachyandesites or trachybasalts) and acidic (porphyries and rhyolites) rocks. Meanwhile, clastic and chemical sediments (i.e. shales, sandstones, and limestones) separate basic and acidic magmatic rock and thus reflect the presence of relatively short intervals that lacked in magmatic activity^31^. Additionally, previous petrological studies (e.g.^21,32,33^) have shown that early-Permian volcanism in the study area was marked by post-collisional and extension-related (continental) affinity.

## Results

### Sample description

**Figure 3:**
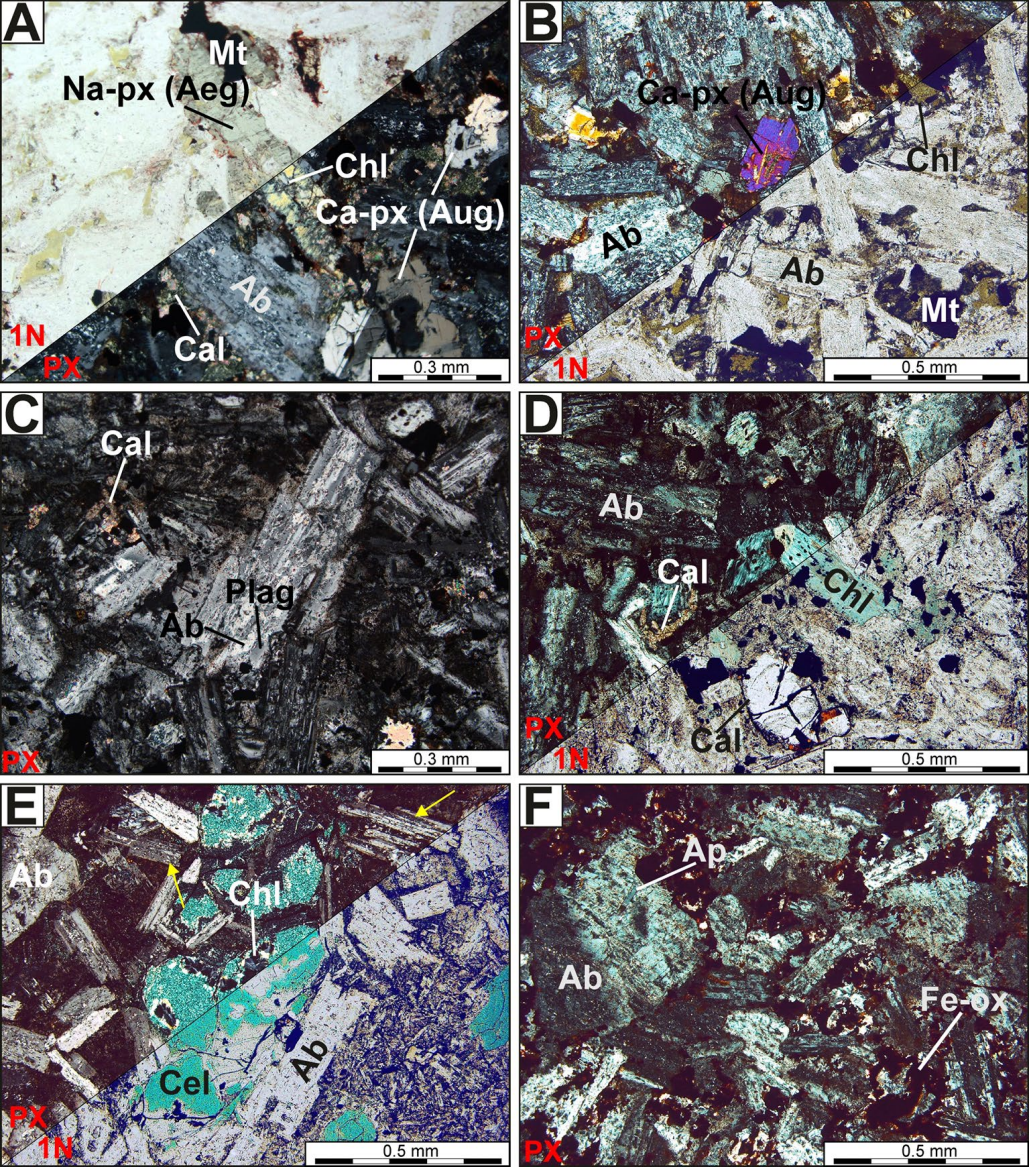
Photomicrographs of trachyandesites from Głuszyca revealing variable alteration style and degree. (**A**) Partially-chloritized Na-rich clinopyroxene (aegirine; Aeg) accompanied by twinned Ca-rich clinopyroxene (augite; Aug) and magnetite (Mt)—Loc. 1 (lower part of the laccolith). (**B**) Ca-rich clinopyroxene (augite) surrounded by partially-albitized (Ab) plagioclase laths and massive to bundle-like (dark-green) chlorite aggregates (Chl)—Loc. 1. (**C**) Incipient stages of albitization accompanied by the formation of calcite (Cal)—Loc. 1. **(D)** Complete replacement of pyroxene by pale-green and fibrous chlorite crystals accompanied by oval-shaped calcite—Loc. 1. (**E**) Celadonite (Cel, marked by intense green colouration that partially masks original int. colours) intergrown with chlorite (Chl; colourless, with anomalous bluish-gray int. colours), both embedded within rock matrix consisting of entirely albitized plagioclase laths surrounded by aphanitic volcanic glass (porphyric micro-texture)—Loc. 2; note the presence of original twinning planes in secondary albite inherited from magmatic precursor (yellow arrow). (**F**) Albitized plagioclases accompanied by numerous opaque to reddish iron oxides (Fe-ox)—Loc. 3. Note the presence of apatite (Ap) enclosed in albitized primary plagioclase.

The samples collected from various parts of the laccolith, i.e. lower (Loc. 1; Fig. 2A), middle (Loc. 2, Fig. 2B), and upper (Loc. 3.; Fig. 2A) have been examined. On the basis of macroscopic features and thin section observations, four types of rock samples marked by variable alteration degrees and style could be distinguished (Table 1; Fig. 3A–F). The pre-existing (original) mineralogical and microtextural features of the rocks were only preserved in samples from the lowermost parts of the magmatic body (samples type GL_01A and GL_01B; Loc. 1). These rocks still contain some relicts of unaltered (magmatic) pyroxenes and plagioclases, as well as opaques (magnetite and ilmenite). The middle (sample type GL_02; Loc 2.) and upper (sample type GL_03; Loc. 3) parts of the laccolith from Głuszyca consist mostly of spilitic-related secondary minerals assemblages, including i.e. albite (after primary plagioclase) and chlorite (either after primary pyroxene or filling vugs and veinlets), accompanied by secondary titanite, calcite, celadonite, and Fe-oxides. Meanwhile, all samples are marked by the presence of accessory apatite.

**Table 1:** Mineralogical and micro-textural description of the four samples types (GL_01A, GL_01B, GL_2, GL_03), which has been distinguished on the basis of alteration style and degree (i.e. chloritization and albitization) and collected from various (i.e. lower—Loc. 1, middle—Loc. 2, and upper—Loc. 3) regions of Głuszyca trachyandesite laccolith.

Sample number	Sample location within the laccolith	Mineralogy		Colour/ micro-texture
		Primary (magmatic) phases	Secondary (hydrothermal) phases	
GL_01A GL_01B*	Lower (Loc. 1)	Magnetite/ilmenite, fluorapatite, pyroxene (augite), hornblende, plagioclase (andesine-labradorite), quartz K-feldspar	albite, chlorite-group minerals, calcite, baddeleyite(?), titanite	Black-coloured/fine-crystalline, sub-ophitic, non-foliated
GL_02	Middle (Loc. 2)	Magnetite/ilmenite, fluorapatite	albite, celadonite, chlorite-group minerals, calcite	Green-coloured/ porphyric (plagioclase embedded within cryptocrystalline groundmass), non-foliated
GL_03	Upper (Loc. 3)	Magnetite/ilmenite, fluorapatite, K-feldspar	albite, chlorite-group minerals Fe-oxides (hematite), calcite, chalcedony, sericite	Red-coloured/fine-crystalline amygdaloidal and/or vesicular, non-foliated

*Note the samples GL_01A and GL_01B were distinguished due to variable albitization and chloritization degree. The former were marked by partial albitization of primary plagioclases and partial chloritization of primary pyroxenes, whereas the latter revealed partial albitization and pervasive chloritization.

### Petrography and mineral geochemistry

#### Pyroxene

**Figure 4:**
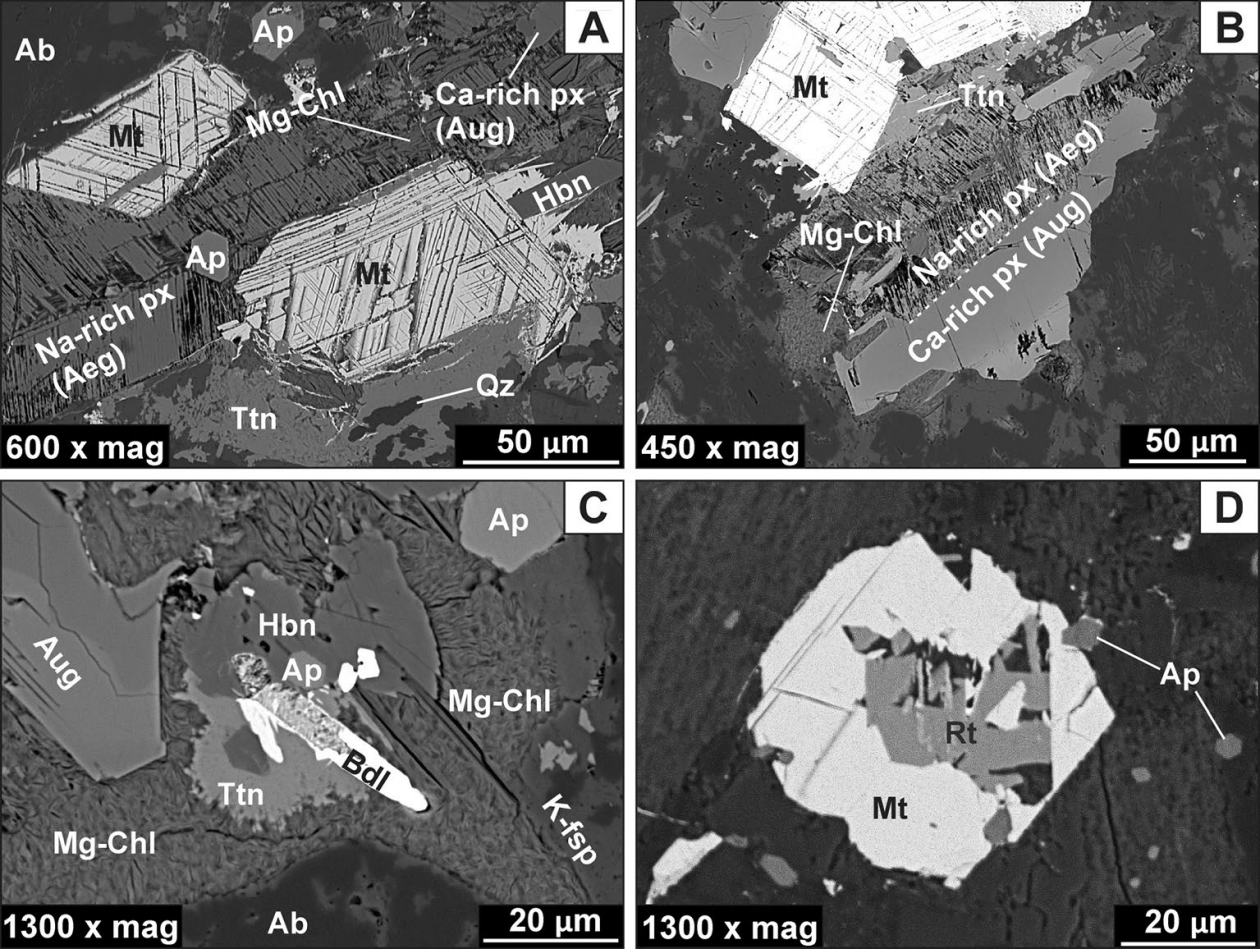
SEM-BSE images of Głuszyca trachyandesites collected from lower parts of the laccolith and showing the presence of unaltered mafic phases such as pyroxene-group species and Ti–rich magnetite. (**A**) Na-rich pyroxene (aegirine) accompanied by Ti–rich magnetite (Mt) consisting oriented thin (trellis-type) intergrowths of ilmenite lamellae. Note the presence of hornblende (Hbn), apatite (Ap), as well as secondary titanite (Ttn) and quartz (Qz); (**B**) Chloritized Na-rich pyroxene (aegirine) enveloped by relatively fresh Ca-rich pyroxene (augite); Note the sharp boundary between those phases, as well as the formation of etched cleavage planes within host Na-rich pyroxene. (**C**) Partial alteration of augite into fibrous Mg-rich chlorite (Mg-Chl); Note the presence of minor hornblende (Hbn), baddeleyite (ZrO_2_; Bdl), and apatite (Ap). (**D**) Alteration of primary Ti–rich magnetite (Mt) into secondary rutile (or anatase—Rt).

Pyroxene occurs as prismatic subhedral crystals up to ca 0.3 mm in size and has been partially (i.e. Loc. 1, sample GL_01A) or almost entirely (samples GL_01B, GL_02, and GL_03) converted into deep-green massive or bundle-like aggregates of chlorites (see: Fig. 3A–B). Locally, the pervasive alteration of pyroxene has also led to the formation of other, fibrous and pale-green chlorite crystals (see: Fig. 3D). SEM-BSE observations revealed that pyroxenes are represented by both Na-rich (1) and Ca-rich (2) species. Both of them are predominately clustered together (i.e. Na-rich pyroxene is rimmed by Ca-rich pyroxene), but also occur in the form of individual crystals (Fig. 4A–B). Meanwhile, the boundary between Na-rich and Ca-rich pyroxenes phases is quite sharp as shown in Fig. 4B.

**Figure 5:**
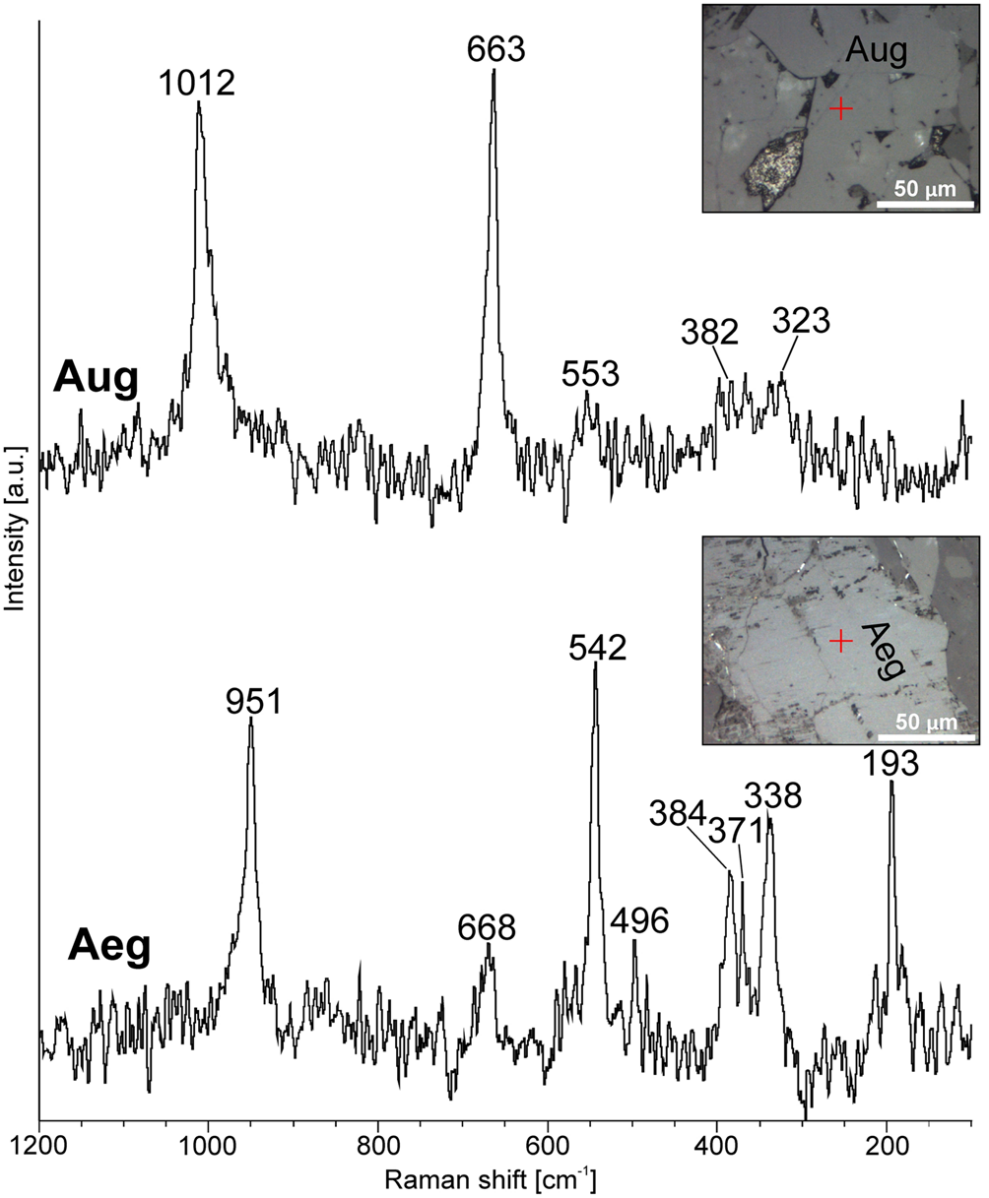
Raman spectra for augite (Aug) and aegirine (Aeg) in the spectral range of 1200–100 cm^−1^ along with corresponding photomicrographs (reflected light mode) and analytical point marked by red cross.

Na-rich (1) pyroxene is marked by relatively low CaO (~ 3.37 wt.%) but elevated Na_2_O (~ 10.56 wt.%) and Fe_2_O_3_ (~ 25.20 wt.%) contents—Table 2. It contains traces of MnO and TiO_2_ (~ 0.36 and ~ 0.45 wt.%, respectively) whilst Mg# values are constant and relatively high as they fall within the range of 0.57–0.77. The average EMPA-based formula of Na-rich pyroxene was calculated as (Na_0.79_Ca_0.14_Fe_0.06_^2+^Mn_0.01_)(Fe_0.74_^3+^Mg_0.18_Fe_0.04_^2+^Al_0.03_Ti_0.01_) (Si_2_O_6_), and corresponds to aegirine based on Q–Jd–Aeg relationship after Morimoto^34^. The presence of aegirine was further confirmed by Raman microspectroscopic measurements (Table 3, Fig. 5). Its diagnostic bands are found at 951 and 542 cm^−1^ and assigned to Si–O_nbr_ and Si–O_br_ stretching vibrations, respectively^35^. Under SEM-BSE images, aegirine often appears riddled as it shows etched cleavage planes, forming so-called chevron (V-shaped) pattern, as well as the presence of co-existing (replacive?) Mg-chlorites. It also occasionally comprises tabular calcite up to ca. 50 µm in size. The aegirine locally form intergrowths with magnetite that not only shows thin oriented trellis-type ilmenite lamellae, but also alters to rutile (or anatase)—Fig. 4A and D. Moreover, aegirine is frequently surrounded by fan-shaped aggregates of titanite followed by minor amounts of quartz.

**Table 2:** Representative clinopyroxene (aegirine and augite) compositions (EMPA) from the samples collected from lower parts of the exposure (Loc. 1) (based on 6 oxygen atoms).

Species	Ca-rich pyroxene (augite)								Na-rich pyroxene (aegirine)							
	Inner areas of the crystal						Outer areas of the crystal									
**Element (wt. %)**																
SiO_2_	51.47	50.25	50.68	51.68	50.75	51.84	50.73	50.58	51.24	51.57	51.59	51.78	51.74	51.81	51.83	51.75
TiO_2_	0.75	0.68	0.69	0.65	0.66	0.74	0.25	0.33	0.48	1.16	0.34	0.24	0.25	0.19	0.71	0.25
Al_2_O_3_	1.02	1.17	1.16	1.16	1.03	1.16	0.58	0.52	0.57	0.67	0.57	0.59	0.55	0.53	0.67	0.58
FeO	15.50	15.13	16.02	15.42	16.33	15.43	20.45	21.85	2.62	3.64	2.03	1.70	3.43	2.11	4.81	2.83
Fe_2_O_3_ (*calc.*)	0.00	0.38	0.87	0.13	0.00	0.00	0.00	0.04	25.48	23.66	25.50	26.19	25.61	26.06	23.42	25.71
MnO	0.35	0.35	0.50	0.42	0.43	0.45	0.64	0.45	0.35	0.34	0.36	0.37	0.37	0.45	0.27	0.36
MgO	12.60	12.65	12.44	13.35	11.18	13.07	9.26	8.79	2.91	3.58	3.17	3.16	2.93	3.13	3.53	3.03
CaO	16.89	16.52	15.79	16.77	16.42	17.11	16.68	16.88	3.35	3.18	3.63	3.50	3.20	3.44	3.38	3.26
Na_2_O	0.22	0.32	0.50	0.27	0.75	0.21	0.22	0.25	10.62	10.41	10.63	10.77	10.56	10.69	10.12	10.64
Total	98.79	97.45	98.63	99.84	97.56	100.01	99.34	99.69	97.62	98.21	97.83	98.30	98.64	98.41	98.73	98.40
**a.p.f.u**																
Si	1.98	1.96	1.96	1.96	1.99	1.97	2.00	1.99	2.00	2.00	2.00	2.00	2.00	2.00	2.00	2.00
Ti	0.02	0.02	0.02	0.02	0.02	0.02	0.01	0.01	0.01	0.03	0.01	0.01	0.01	0.01	0.02	0.01
Al	0.05	0.05	0.05	0.05	0.05	0.05	0.03	0.02	0.03	0.03	0.03	0.03	0.03	0.02	0.03	0.03
Fe^2+^	0.50	0.49	0.52	0.49	0.53	0.49	0.68	0.72	0.09	0.12	0.07	0.06	0.11	0.07	0.16	0.09
Fe^3+^	0.00	0.01	0.03	0.00	0.00	0.00	0.00	0.00	0.75	0.69	0.75	0.76	0.75	0.76	0.68	0.75
Mn	0.01	0.01	0.02	0.01	0.01	0.01	0.02	0.01	0.01	0.01	0.01	0.01	0.01	0.01	0.01	0.01
Mg	0.72	0.74	0.72	0.76	0.65	0.74	0.54	0.51	0.17	0.21	0.18	0.18	0.17	0.18	0.20	0.17
Ca	0.70	0.69	0.65	0.68	0.69	0.70	0.71	0.71	0.14	0.13	0.15	0.15	0.13	0.14	0.14	0.14
Na	0.02	0.02	0.04	0.02	0.06	0.02	0.02	0.02	0.80	0.78	0.80	0.81	0.79	0.80	0.75	0.80
#Mg	0.59	0.59	0.58	0.61	0.55	0.60	0.44	0.42	0.65	0.64	0.72	0.77	0.60	0.73	0.57	0.66
Q	1.92	1.92	1.89	1.93	1.88	1.93	1.93	1.94	0.40	0.46	0.40	0.38	0.41	0.39	0.50	0.40
J	0.03	0.05	0.07	0.04	0.11	0.03	0.03	0.04	1.60	1.56	1.60	1.62	1.58	1.60	1.50	1.60
Q + J	1.95	1.97	1.96	1.97	1.99	1.96	1.96	1.98	2.00	2.00	2.00	2.00	1.99	1.99	2.00	2.00
J/(Q + J)	0.02	0.02	0.04	0.02	0.06	0.02	0.02	0.02	0.80	0.77	0.80	0.81	0.79	0.80	0.75	0.80
Wo	36.31	35.97	34.62	35.39	36.72	36.15	36.75	36.56	–	–	–	–	–	–	–	–
En	37.68	38.32	37.96	39.21	34.79	38.42	28.04	26.49	–	–	–	–	–	–	–	–
Fs	26.00	25.71	27.42	25.40	28.50	25.43	35.20	36.94	–	–	–	–	–	–	–	–
Quad	–	–	–	–	–	–	–	–	14.39	16.79	14.39	13.72	14.91	14.10	18.29	14.47
Jd	–	–	–	–	–	–	–	–	29.86	29.56	29.86	29.94	29.53	29.73	28.92	29.75
Aeg	–	–	**–**	–	–	–	–	–	55.76	53.65	55.76	56.34	55.56	56.18	52.79	55.78

Note: #Mg = 100 Mg/(Mg + Fe^2+^); Fe^3+^ calculated from charge balance; Q = Ca + Mg + Fe^2+^; J = 2Na.

**Table 3:** Representative composition (EMPA) of greenish-gray luminescent primary plagioclase and secondary (weak-luminescent to dark-brown luminescent) replacive albite (based on 8 oxygen atoms).

Pyroxene type	Raman bands [cm^−1^]	Assignment*
Augite	1012(s)	Stretching modes of Si–O_nb_
	663(s)	Stretching modes of Si–O_br_
	553(w)	O–Si–O bending modes
	382(w), 323(w)	Cation-oxygen vibrations
Aegirine	951(s)	Stretching modes of Si–O_nb_
	668(w), 542(s)	Stretching modes of Si–O_br_
	496(w)	O–Si–O bending modes
	384(s), 371(s), 338(s), 193(s)	Cation-oxygen vibrations

*After i.e.^35^; _nb_—non-bridging modes, _br_—bridging modes; s and w correspond to strong and weak intensity diagnostic Raman bands.

Conversely, Ca-rich pyroxene (2) is relatively depleted in Na_2_O (~ 0.34 wt.%), but rich in CaO (~ 11.67 wt.%), with average formula of (Ca_0.69_Fe_0.27_^2+^Na_0.03_Mn_0.01_) (Mg_0.67_Fe_0.28_^2+^Al_0.02_Ti_0.02_Fe_0.01_^3+^)(Si_1.98_Al_0.02_ O_6_) typical of augite species (Table 2). It comprises minor amounts of MnO and TiO_2_ (~ 0.45 and ~ 0.59 wt.%, respectively). Mg#(molar Mg/Mg + Fe) values are variable and range between 0.55 and 0.61 (inner areas of the crystals) and 0.42–0.44 (outer regions of the crystals). The Raman spectrum of augite is shown in Fig. 5 (see also Table 3) and comprises two strong marker bands at 1012 and 663 cm^1^ attributed to Si–O_nbr_ and Si–O_br_ stretching modes^35^. Augite occasionally exhibits oscillatory zonation under SEM-BSE images maintained by the variations in Fe and Mg contents. Additionally, it is accompanied by minor amounts of hornblende, as well as minute elongated crystals of baddeleyite (ZrO_2_)—Fig. 4C. The breakdown of augite into chlorite has also been noted, although in the vast majority of investigated samples the pervasive chloritization has totally wiped out original microtextural features of the rocks and, thus the detailed identification of pre-existing pyroxene is not available.

#### Feldspar-group minerals

**Figure 6:**
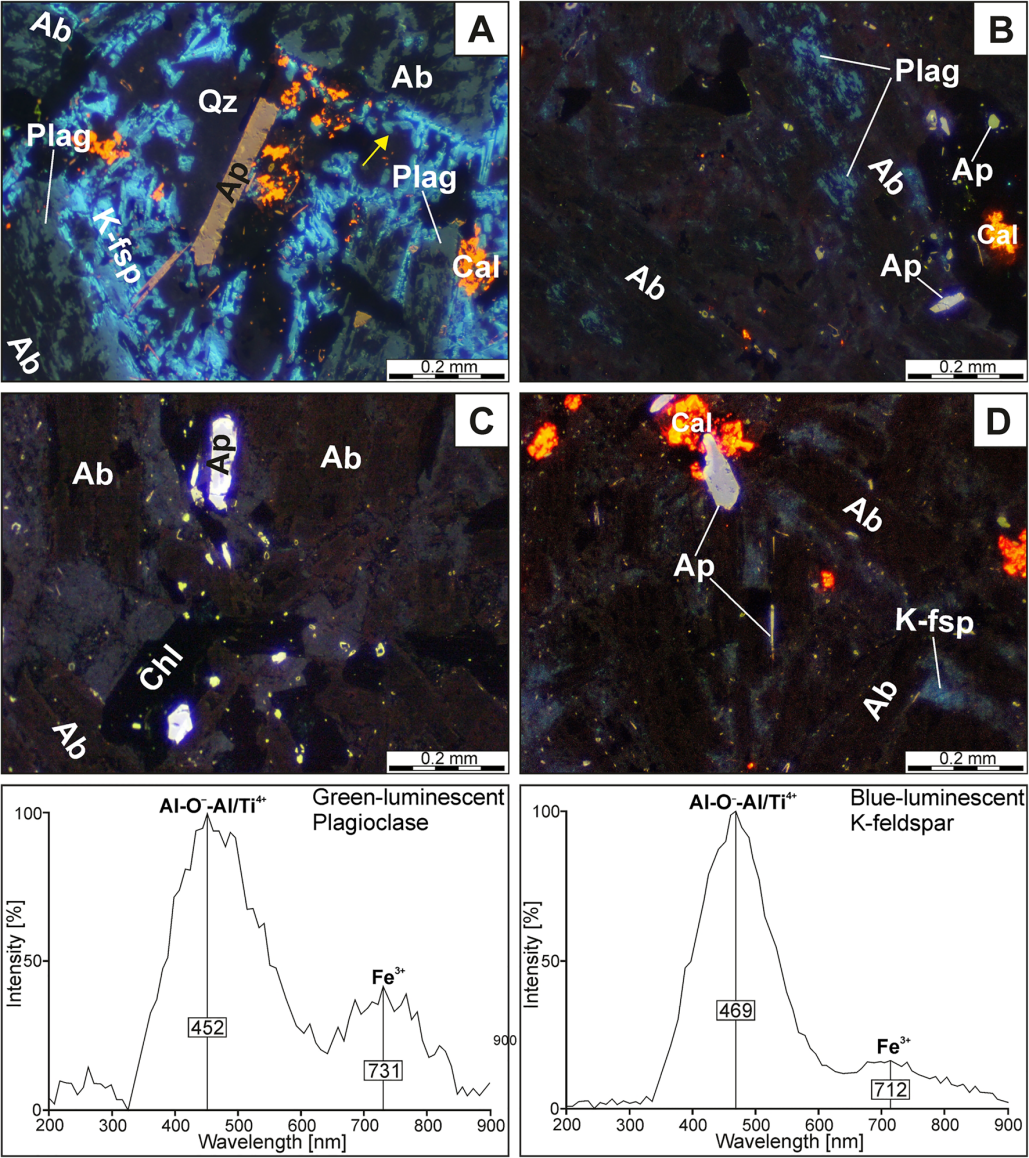
Variable stages of albitization developed within Głuszyca trachyandesites revealed using OM-CL technique. (**A**) Incipient albitization marked by the occurrence of pure, weak to dark-brown luminescent albite (Ab) patches invading primary greenish-gray luminescent plagioclase host (andesine-labradorite) (Plag); Note that albitized andesine-labradorite is rimmed by relatively fresh blue-luminescent K-feldspar (K-fsp) showing embayed grain contacts with interstitial weakly-luminescent quartz (yellow arrow)—Loc. 1. (**B**) Progressive albitization of primary andesine-labradorite (Plag) (Loc. 1). (**C, D**) Complete replacement of andesine-labradorite by brown-luminescent secondary albite (Ab) in the samples from the middle (Loc. 2) and upper (Loc. 3) parts of the laccolith from Głuszyca, respectively. Note that CL spectra of greenish-gray luminescent plagioclase and bright-blue luminescent K-feldspar in the spectral range of 200–900 nm were shown at the bottom of the image.

Primary plagioclase that has been partially preserved from pervasive albitization was recognized only in the samples collected from the lowermost parts of the laccolith (Loc. 1)—see Fig. 6A and B. It forms equigranular lath-shaped crystals (up to 0.4 mm in size) with greenish, greenish-grey, and/or bluish luminescence. The CL spectrum of primary plagioclase comprises a broad (blue) band centred at 452 nm, accompanied by the weak-intensity peak at ca. 731 nm. The former band could be attributed to either Al-O^–^-Al centres, related to the coupled K-Si–Ba-Al substitution, or Al-O-Ti bridges, or both defects^21,36^, whereas the latter band corresponds to traces of Fe^3+^ in the mineral structure. Partially and/or completely albitized plagioclase is frequently rimmed by unaltered and bright-blue to dark-blue luminescent K-feldspars (Fig. 6A). The CL spectrum of K-feldspar is fairly similar to this collected from green-luminescent plagioclase, since it consists of the strong band at ca. 469 nm (Al-O^–^-Al centres or Ti^4+^ impurity) and negligible (Fe^3+^-related) band at ca. 712 nm. K-feldspars are followed by interstitial (magmatic) quartz that exhibits weak (dark-blue) CL colours and embayed crystal margins, as shown in Fig. 6A.

Green and/or greenish-gray luminescent plagioclase is marked by intermediate composition, i.e. An contents range from 37.33 to 50.49 mol.%, whilst Ab concentration is between 46.85 and 58.38 mol.% (Table 4). Thus, it represents either andesine or labradorite members of the feldspar group. Primary plagioclase also contains trace amounts of TiO_2_ (~ 0.09 wt.%) and MgO (~ 0.09 wt.%), and K_2_O (0.49 wt.%).

**Figure 7:**
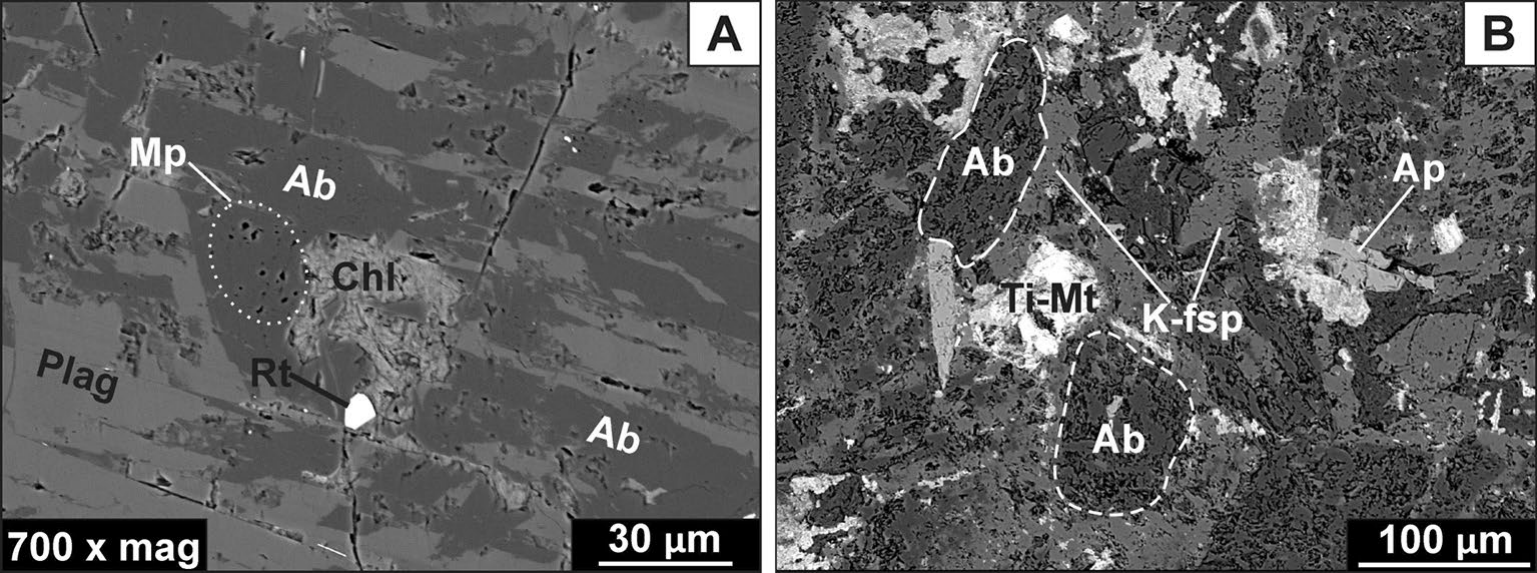
SEM-BSE images showing albitization in trachyandesites from Głuszyca. (**A**) Partial albitization of primary plagioclase (andesine-labradorite) followed by the formation of minor rutile; note the secondary albite do not contain any visible cogenetic inclusions except for chlorite that could precipitate in voids (Loc. 1); (**B**) Complete albitization of andesine-labradorite (Loc. 3); Note the presence K-feldspar surrounding albitized andesine-labradorite, as well as abundant micron-sized micropores developed within the secondary albite.

The pervasive albitization of trachyandesites from Głuszyca (see Fig. 6C and D) has resulted in the presence of almost chemically pure (Ab ~ 99 mol.%—Table 4) secondary albite that forms elongated patches penetrating through host magmatic andesine-labradorite and displays weak to dark-brown cathodoluminescence (Fig. 6C and D). The albite occurs as non-twinned crystals with “dusty” appearance in thin sections, but original twinning of primary plagioclase (andesine-labradorite) seems to be only locally preserved in completely albitized samples (Fig. 3E). The contact between secondary albite and andesine-labradorite is sharp on the micrometer scale, as shown in the BSE images (Fig. 7A). Furthermore, albitization of andesine-labradorite was accompanied by the emergence of numerous micropores (Fig. 7A and B) and the occasional formation of minute rutile (or anatase) crystals.

**Table 4:** Representative composition (EMPA) of fluorapatite from the samples with variable alteration degrees (based on 26 oxygens following the procedure proposed by^85^.

Type	Primary plagioclase					Secondary (replacive) albite				
CL colours	Greenish to greenish-gray					Weak-luminescent to dark-brown				
**Element wt. %**										
SiO_2_	55.00	57.68	55.00	54.65	54.59	67.44	67.40	67.46	67.64	67.84
TiO_2_	0.08	0.09	0.10	0.10	0.09	0.00	0.00	0.00	0.00	0.00
Al_2_O_3_	27.16	25.14	26.93	27.41	27.11	19.43	19.34	19.52	19.29	19.59
Fe_2_O_3_*	0.12	0.21	0.00	0.00	0.60	0.15	0.07	0.02	0.03	0.14
FeO	0.38	0.31	0.57	0.55	0.00	0.00	0.00	0.05	0.00	0.00
MgO	0.09	0.09	0.06	0.09	0.10	0.00	0.00	0.00	0.00	0.00
CaO	9.72	7.62	9.80	10.17	9.86	0.05	0.08	0.13	0.06	0.06
Na_2_O	5.56	6.58	5.41	5.21	5.44	11.77	11.82	11.50	11.72	11.71
K_2_O	0.35	0.74	0.46	0.45	0.45	0.00	0.02	0.04	0.04	0.05
Total	98.46	98.46	98.33	98.62	98.23	98.85	98.72	98.71	98.79	99.38
**Formula based on O = 8**										
Si	2.52	2.63	2.52	2.50	2.51	2.98	2.98	2.98	2.99	2.98
Ti	0.00	0.00	0.00	0.00	0.00	0.00	0.00	0.00	0.00	0.00
Al	1.46	1.35	1.46	1.48	1.47	1.01	1.01	1.02	1.00	1.01
Fe^3+^	0.00	0.01	0.00	0.00	0.02	0.01	0.00	0.00	0.00	0.00
Fe^2+^	0.01	0.01	0.02	0.02	0.00	0.00	0.00	0.00	0.00	0.00
Mn	0.00	0.00	0.00	0.00	0.00	0.00	0.00	0.00	0.00	0.00
Mg	0.01	0.01	0.00	0.01	0.01	0.00	0.00	0.00	0.00	0.00
Ca	0.48	0.37	0.48	0.50	0.49	0.00	0.00	0.01	0.00	0.00
Na	0.49	0.58	0.48	0.46	0.48	1.01	1.01	0.99	1.00	1.00
K	0.02	0.04	0.03	0.03	0.03	0.00	0.00	0.00	0.00	0.00
Cation sum	5.0	5.0	5.0	5.0	5.0	5.0	5.0	5.0	5.0	5.0
**End member proportions**										
An	48.15	37.33	48.68	50.49	48.72	0.22	0.36	0.61	0.29	0.27
Ab	49.78	58.38	48.61	46.85	48.66	99.75	99.56	99.19	99.48	99.46
Or	2.07	4.29	2.70	2.67	2.62	0.02	0.08	0.20	0.23	0.26

The crystals oriented with their *c*-axes perpendicular to the incident electron beam were selected for the analyses to reduce the accumulation of halogens close to the mineral surface (see e.g.^86^). However, a slight and local excess of halogens (> 2.0 a.p.f.u.) was occasionally presented. The areas showing both yellowish and pinkish CL colours have been investigated individually, but they turned to present a similar distribution of particular elements.

*Note that Fe_2_O_3_ was calculated from charge balance.

#### Fluorapatite

**Figure 8:**
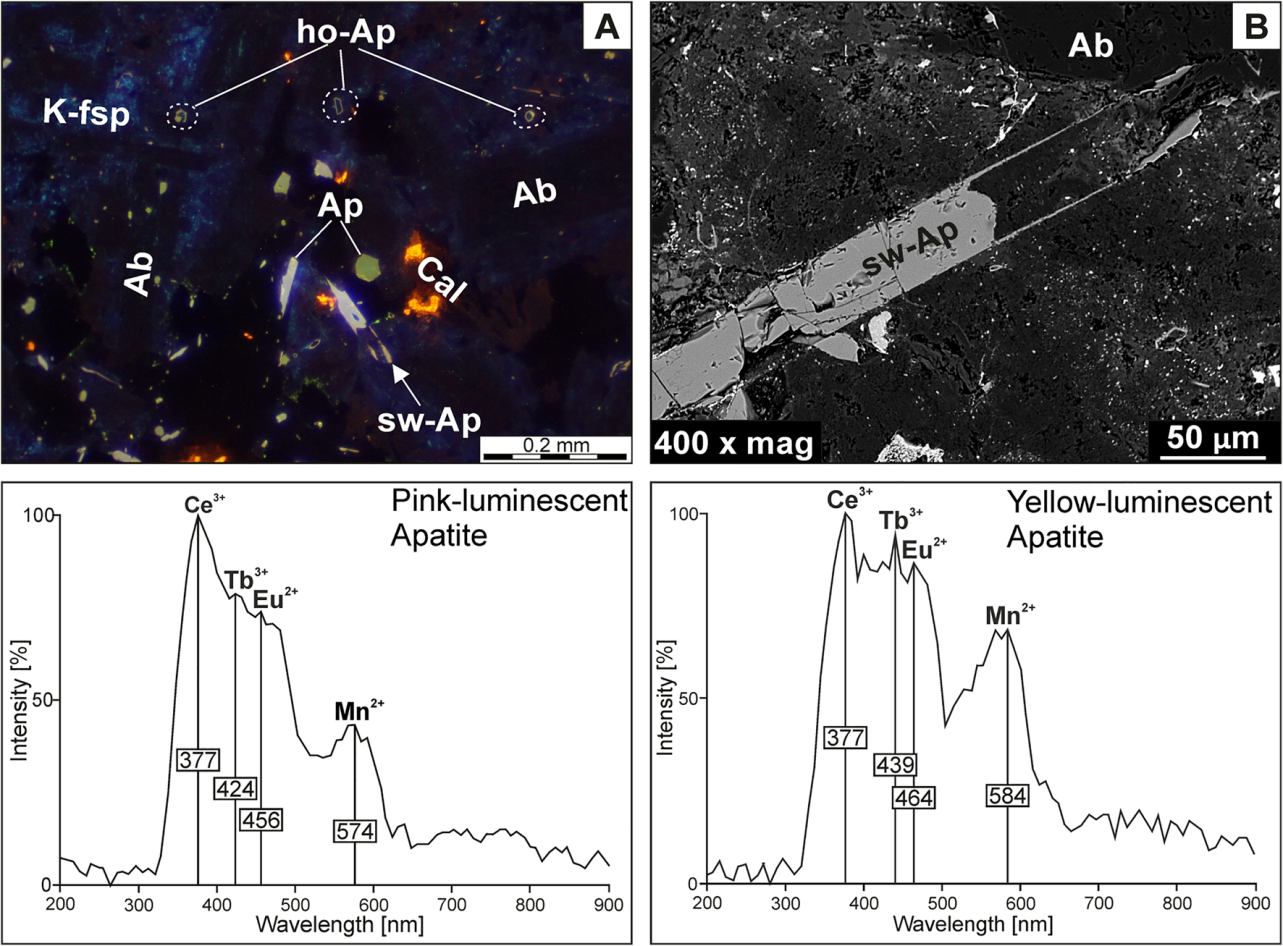
(**A**) Two-types of fluorapatite species showing pinkish and yellowish CL colours depending on size and orientation of particular crystals (Loc. 1); Note the occurrence of hollow-type (ho-Ap) and swallow-tail (sw-Ap) fabrics. (**B**) BSE image of fluorapatite (embedded within the cryptocrystalline groundmass) showing well-developed swallow-tail morphology (Loc. 2). Note the CL spectra of pink-luminescent and yellow-luminescent fluorapatite in the spectra range 200–900 nm were included at the bottom of the image.

Fluorapatite forms euhedral or subhedral acicular crystals showing quite large variations in size (from several µm up to *ca.* 0.4 mm). It exhibits pinkish and/or greyish-blue luminescence (Fig. 8A), which is well-visible in prismatic sections. In contrast, tiny apatite crystals, as well as those situated perpendicular to the c-axis in thin sections display yellowish CL colours. A discrete yellow-luminescent glow has also developed along the margins of particular larger crystals. CL observations revealed that fluorapatite locally exhibits skeletal hollow-type fabrics characterized by the preservation of crystal outlines (Fig. 8A), with their interior infilled by other phases such as secondary albite. Additionally, few crystals revealed the presence of H-shaped (swallow-tail) terminations (Fig. 8B). The CL spectrum of pink-luminescent fluorapatite is marked by strong lines at 377, 424, and 456 nm. These bands correspond to the trace amounts of REE^3+^ within apatite structure^37^ including Ce^3+^ and Tb^3+^, as well as Eu^2+^, respectively. Additionally, the low-intensity band located at 574 nm is likely related to the traces of Mn^2+^^37^. The spectrum of yellow-luminescent apatite is similar to the one recorded for apatite with pink CL colour, as its strongest (REE^3+^-related) lines were observed at 377 (Ce^3+^-activator) and 439 (Tb^3+^-activator), along with 464 nm (Eu^2+^-activator). However, the Mn^2+^-related band found at 584 nm displays quite higher intensity relative to the corresponding line from pink-luminescent apatite.

Fluorapatite displays lack of chemical zonation in BSE images (Fig. 8B). The compositional variations of apatite from various parts of trachyandesite exposure are presented in Table 5. The crystals do not show notable variations in P_2_O_5_ and CaO contents, which range between 39.79 and 41.85 and 53.71–55.29 wt.%, respectively. Otherwise, [∑REE + Y] contents are relatively high (~ 1.03 wt.%) in fluorapatite from lower parts of the laccolith (i.e. sample GL_01A), while the crystals from the samples showing pronounced secondary alterations (i.e. chloritization and albitization) contain ~ 0.50 wt% (sample GL_01B), ~ 0.42 wt% (sample GL_02), and ~ 0.45 wt% (sample GL_02) of [∑REE + Y]. Overall, fluorapatite contain traces of MnO (up to 0.14 wt. %), SiO_2_ (up to 0.37 wt.%), and FeO (up to 0.88 wt.%). The abundances of volatiles (F, Cl, and OH) occur in a narrow range excluding a slight increase in Cl content (~ 0.37 wt.%) in rock samples with porphyric microtextures (sample GL_02). Meanwhile, the fluorapatite shows a strong predominance of F over Cl, as F/Cl ratios are within the range of 8.04–19.95. The content of OH (calculated from stoichiometry, i.e. assuming F + OH + Cl = 2) is very low and varies between 0 and0.10 wt. %, while SO_3_ is below detection limits.

**Table 5:** Position and assignment of Raman bands for co-existing augite and aegirine.

Sample	Locality 1 (lower part of the laccolith)				Locality 2 (middle part of the laccolith)		Locality 3 (upper part of the laccolith)	
Microtexture	Holocrystalline, fine-crystalline				Porphyric		Holocrystalline	
Alterations	Partially-altered **cpx**, and partially-altered **plag**		Complete chloritization of **cpx**, partial albitization of **plag**,		Pervasive chloritization of **cpx** and albitization of **Ca-rich plag** ,celadonitization of volcanic glass		Pervasive albitization of **Ca-rich plag**., chloritization of **px** and intense hematitization (after **Mt** and **Ilm**)	
Anal. points	8		8		8		8	
Sample	GL-01A		GL-01B		GL-02		GL-03	
Element (wt.%)	Av.(*min;max*)	σ	Av. (*min;max*)	σ	Av.(*min; max*)	σ	Av.(*min; max*)	σ
P_2_O_5_	40.86 (39.85;41.78)	0.57	41.12 (40.30;41.85)	0.49	40.46(39.79;41.09)	0.42	40.78 (39.94;41.30)	0.46
SiO_2_	0.24 (0.16;0.33)	0.05	0.22 (0.16;0.37)	0.07	0.21 (0.17;0.24)	0.03	0.20 (0.15;0.28)	0.04
CaO	54.67 (54.00;55.29)	0.43	54.12 (53.71;54.34)	0.22	54.47(54.08;54.29)	0.37	54.55 (54.21;55.07)	0.29
MnO	0.04 (0.00; 0.13)	0.04	0.02 (0;0.14)	0.05	0.03 (.000;0.14)	0.06	0.01 (0.00;0.10)	0.04
FeO	0.48 (0.33;0.62)	0.10	0.55 (0.31;0.88)	0.16	0.52 (0.44;0.61)	0.05	0.54 (0.45;0.76)	0.10
MgO	0.01 (0.00;0.14)	0.04	0.20(0.00;0.25)	0.08	0.17(0.05;0.24)	0.06	0.16 (0.00;0.29)	0.11
Na_2_O	0.11 (.000;0.18)	0.05	0.02(0.00;0.10)	0.04	0.03(0.00;0.07)	0.03	0.02(0.00;0.07)	0.03
La_2_O_3_	0.20 (0.00;0.37)	0.15	0.12(0.00;0.23)	0.10	0.02(0.00;0.17)	0.06	0.08 (0.00;0.25)	0.11
Ce_2_O_3_	0.44 (0.19;0.59)	0.13	0.23(0.15;0.47)	0.11	0.24(0.18;0.34)	0.06	0.24 (0.17;0.42)	0.09
Nd_2_O_3_	0.21 (0.00;0.35)	0.12	0.07(.000;0.32)	0.11	0.05(0.00;0.15)	0.07	0.03 (0.00;0.14)	0.06
Y_2_O_3_	0.11 (.000;0.17)	0.05	0.04(0.00;0.14)	0.05	0.06(0.00;0.11)	0.05	0.05 (0.00;0.11)	0.05
∑REE + Y	1.03 (0.34;1.60)	0.50	0.50 (0.23;1.23)	0.32	0.42 (0.18;0.80)	0.21	0.45 (0.24;0.72)	0.17
Cl	0.24 (0.20;0.27)	0.02	0.32(0.26;0.35)	0.03	0.37(0.34;0.41)	0.02	0.30 (0.25;0.38)	0.04
F	3.84 (3.66;4.09)	0.15	3.55(3.38;3.71)	0.12	3.57 (3.32;4.20)	0.27	3.96 (3.72;4.38)	0.24
Total	99.82 (99.13;100.68)	0.64	99.32 (98.43;100)	0.50	98.96 (98.38;100.81)	0.82	99.52 (98.42;100.33)	0.57
–O = (F + Cl)	1.67 (1.59;1.78)	0.07	1.58 (1.50;1.65)	0.06	1.59 (1.49;1.85)	0.11	1.74 (1.65;1.91)	0.10
H_2_O *(calc.)*	–	–	0.03 (0.00; 0.14)	0.05	0.02 (0.00;0.10)	0.04	–	–
**a.p.f.u**								
P	5.90 (5.81;5.97)	0.05	5.94 (5.89;6.01)	0.03	5.89 (5.85;5.92)	0.02	5.91 (5.86;5.94)	0.03
Si	0.04 (0.03;0.06)	0.01	0.04 (0.03;0.06)	0.01	0.04 (0.03;0.04)	0.02	0.03 (0.03;0.05)	0.01
Ca	9.99 (9.88;10.20)	0.09	9.89 (9.76;10.05)	0.08	10.04 (9.98;10.12)	0.04	10.00 (9.90;10.12)	0.08
Mn	0.00 (.000;0.02)	0	0.00 (0.00;0.02)	0.01	0.00 (0.00;0.02)	0.01	0.00 (0.00;0.01)	0.01
Fe	0.07 (0.05; 0.09)	0.01	0.08 (0.04;0.13)	0.02	0.07 (0.06;0.09)	0.01	0.08 (0.06;0.11)	0.01
Mg	0.00 (0.00;0.03)	0.01	0.05(0.00;0.06)	0.02	0.04 (0.06;0.09)	0.01	0.04 (0.00;0.07)	0.03
Na	0.03 (0.00;0.06)	0.02	0.01 (0.00;0.03)	0.01	0.01 (0.00;0.02)	0.01	0.01 (0.00;0.02)	0.01
La	0.01 (.000;0.02)	0.01	0.01 (0.00;0.01)	0.01	0.00 (0.00; 0.01)	0.00	0.00 (0.00; 0.02)	0.00
Ce	0.03 (0.01;0.04)	0.01	0.01 (0.01;0.03)	0.01	0.02 (0.01;0.02)	0.00	0.01(0.01;0.03)	0.01
Nd	0.01 (0.00;0.02)	0.01	0.00 (0.00;0.02)	0.01	0.00 (0.00;0.01)	0.00	0.00 (0.00;0.01)	0.00
Y	0.01 (0.00;0.02)	0	0.00 (0.00;0.01)	0	0.01 (0.00;0.01)	0.00	0.00 (0.00;0.01)	0.00
**F**	2.07 (1.97;2.18)	0.08	1.92 (1.82;2.01)	0.07	1.94 (1.82;2.25)	0.13	2.14 (2;2.35)	0.12
**Cl**	0.07 (0.06;0.08)	0.02	0.09 (0.08;0.10)	0.01	0.11 (0.10;0.12)	0.01	0.09 (0.07;0.11)	0.01
**OH**	–	–	0.02 (0.00;0.08)	0.03	0.01 (0.00; 0.10)	0.04	–	–

Note: Cpx—clinopyroxene (augite and aegirine); plag—primary plagioclase (andesine-labradorite).

#### Chlorite

**Figure 9:**
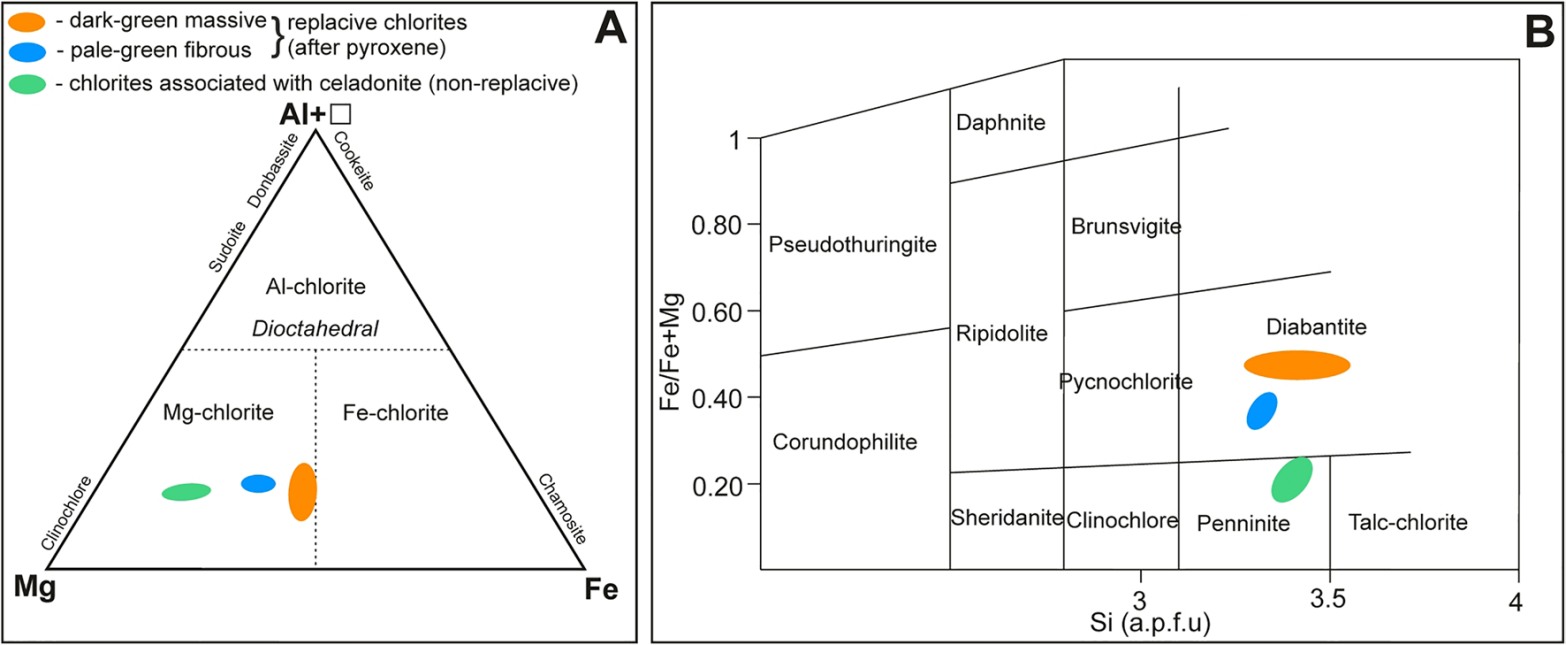
(**A**) Al + □–Mg–Fe compositional classification diagram for replacive chlorites and chlorites associated with celadonite (non-replacive)—after ^38^. Note that □ corresponds to the octahedral vacancy. (**B**) Fe/(Fe + Mg) versus Si (in a.p.f.u.) classification plot of replacive chlorites and non-replacive chlorites associated with celadonite—after ^39^.

Under plane-polarized light, three types of chlorite-group species were distinguished in volcanics: (1) replacive (after pyroxene-group minerals) dark-green aggregates exhibiting massive and bundle-like fabrics, (2) replacive (after pyroxene-group minerals) pale-green fibrous crystals, as well as (3) non-replacive, vugs-filling (colourless) crystals embedded within celadonite. Their compositions are presented in Table 6. The chlorite of the former variety (1) is marked by trioctahedral nature and shows scattered distribution of alkali contents (∑CaO + K_2_O + Na_2_O) in the range of 0.27–1.41 wt.%. Based on Al–Fe–Mg compositional diagram (Fig. 9A see^38^), it falls within the field of Mg-rich chlorites and could be classified as *diabantite* according to [Fe/Fe + Mg]–Si classification plot (Fig. 9B; see^39^). The species are rich in Si showing constant amounts in the range of 3.30–3.54 a.p.f.u. Conversely, Al^IV^ occurs in a range of 0.46–0.70 a.p.f.u and shows depletion relative to Al^VI^ (0.71–0.91). Fe/Fe + Mg ratios are relatively high and range from 0.45 to 0.50. The sum of octahedral cations (5.60–5.93 a.p.f.u.) is variable, whereas octahedral vacancies fall between 0.40 to 0.07 a.p.f.u. The second type of chlorite (2) displays a similar chemical composition to the first type, typical of *diabantite*. Likewise, it is rich in Si (~ 3.32 a.p.f.u.) and shows quite constant amounts of Al^IV^ within the range of 0.65–0.71, as well as high Al^VI^/Al^IV^ ratios (~ 1.43). Conversely, alkali contents (∑CaO + K_2_O + Na_2_O) are low and do not exceed 0.40 wt.%. Mg (~ 3.01 a.p.f.u.) is notably higher than Fe (~ 1.81 a.p.f.u.) whereas Fe/Fe + Mg ratios are in the range of 0.34–0.40. The sum of octahedral cations and octahedral vacancies falls between 5.78–5.83 and 0.22–0.17 a.p.f.u., respectively. Non-replacive chlorite (3), found in the close spatial association with celadonite, is marked by nearly full octahedral occupancy (~ 5.84 a.p.f.u.), and displays minor variations in Si contents (3.36–3.43 a.p.f.u.). Alkali contents (∑CaO + K_2_O + Na_2_O) are low and range between 0.07–0.37 wt.%. Al^VI^ (~ 0.88 a.p.f.u.) occurs in the narrow range and predominates over Al^IV^ (~ 0.61 a.p.f.u.). Non-replacive chlorite is also significantly enriched in Mg (~ 3.99 a.p.f.u.) and depleted in Fe^2+^ (~ 0.94 a.p.f.u.), hence exhibits relatively low but quite variable Fe/Fe + Mg ratios (0.17–0.25). According to [Fe/Fe + Mg]–Si compositional diagram, this chlorite-group species predominately represents *penninite* series—Fig. 9B.

**Table 6:** Representative composition (EMPA) of replacive chlorite (after aegirine and/or augite) and chlorites associated with celadonite (based on 14 oxygen atoms).

Chlorite type	Replacive chlorite (after pyroxene)				Non-replacive, colourless and radiating crystals associated with celadonite	
	Massive, dark-green, granular and/or bundle-like aggregates		Fibrous, pale-green (locally porous) crystals			
Analytical points	14		17		16	
Element (wt.%)	Av. (*min; max*)	σ	Av. (*min; max*)	σ	Av. (*min; max*)	σ
SiO_2_	32.15 (30.17; 33.26)	1.01	31.95 (31.32; 32.51)	0.30	34.34 (33.84; 34.58)	0.22
TiO_2_	0.02 (0; 0.09)	0.02	0.04 (0; 0.08)	0.02	0.05 (0.02; 0.08)	0.01
Al_2_O_3_	11.10 (10.81; 11.33)	0.17	13.46 (12.86; 14.07)	0.27	12.82 (12.52; 13.14)	0.18
FeO	26.12 (24.78; 27.31)	0.89	20.84 (19.34; 22.01)	0.62	11.35 (10.22; 14.54)	1.31
MnO	0.18 (0.15; 0.24)	0.03	0.16 (0.08; 0.24)	0.04	0.25 (0.20; 0.36)	0.04
MgO	16.12 (15.04; 17.45)	0.81	19.40 (18.67; 21.01)	0.58	27.13 (24.27; 28.51)	1.08
CaO	0.69 (0.27; 1.20)	0.32	0.30 (0.27; 0.34)	0.02	0.16 (0.07; 0.33)	0.08
Na_2_O	0.03 (0; 0.15)	0.05	0.01 (0; 0.06)	0.02	–	–
K_2_O	0.05 (0; 0.16)	0.05	0.01 (*0; 0.05*)	0.02	0.01 (0; 0.05)	0.02
F	0.03 (0;0.21)	0.07	–	–	–	
Cl	0 (*0; 0.03*)	0.01	0.01 (*0; 0.03*)	0.01	0 (0; 0.01)	0
Totals	86.49 (85.22; 89.16)	0.99	86.17 (84.41; 87.21)	0.77	86.11 (85.59; 87.19)	0.41
Si	3.44 (3.30; 3.54)	0.08	3.32 (3.29; 3.35)	0.01	3.39 (3.36;3.43)	0.02
Al^IV^	0.56 (0.46; 0.70)	0.08	0.68 (0.65; 0.71)	0.01	0.61 (0.57; 0.64)	0.02
Al^VI^	0.84 (0.71; 0.95)	0.07	0.97 (0.94; 1.02)	0.02	0.88 (0.86; 0.91)	0.01
Ti	0 (0; 0.01)	0	0 (0; 0.01)	0	0 (0;0.01)	0
Fe	2.34 (2.22; 2.50)	0.09	1.81 (1.65; 1.91)	0.06	0.94 (0.83; 1.22)	0.11
Mn	0.02 (0.01; 0.02)	0	0.01 (0.01; 0.02)	0	0.02 (0.02; 0.03)	0
Mg	2.57 (2.41; 2.78)	0.12	3.01 (2.91; 3.20)	0.07	3.99 (3.64; 4.14)	0.14
Ca	0.08 (0.03; 0.14)	0.04	0.03 (0.03; 0.04)	0	0.02 (0.01; 0.04)	0.01
Na	0.01 (0; 0.03)	0.01	0 (0; 0.01)	0	–	–
K	0.01 (0; 0.02)	0.01	0 (0; 0.01)	0	0 (0; 0.01)	0
Σ Oct	5.77 (5.60; 5.93)	0.11	5.81 (5.78; 5.83)	0.01	5.84 (5.80; 5.87)	0.02
□	0.23 (0.07; 0.40)	0.11	0.19 (0.17; 0.22)	0.01	0.16 (0.13; 0.20)	0.02
R^2+^	4.93 (4.65; 5.23)	0.18	4.84 (4.76; 4.89)	0.03	4.95 (4.90; 5.00)	0.03
Fe/Fe + Mg	0.48 (0.45; 0.50)	0.01	0.38 (0.34; 0.40)	0.01	0.19 (0.17; 0.25)	0.02
Variety	Mg-chlorite (diabantite)		Mg-chlorite (diabantite)		Mg-chlorite (penninite)	

Note: Al^IV^ = 4—Si^IV^ a.p.f.u.; ΣT: tetrahedral occupancy (Si + Al^IV^); ΣOct: octahedral occupancy (Al^VI^ + Fe + Mg + Mn); □: octahedral vacancy; R^2+^: sum of divalent cations (Fe + Mg + Mn) assuming all iron to be ferric (Fe^2+^).

### Chlorite geothermometry

The temperature of chlorite crystallization has been assessed using five independent empirical geothermometers (Table 7). The temperatures obtained using these methods depend directly on the chemical composition of chlorites and take into account the contents of Si^40^, Al^IV^^41^, as well as combined Al^IV^ content and Fe/Fe + Mg ratios^42–44^. The semi-empirical (graphical) approach proposed by Bourdelle and Cathelineau ^45^, has been also applied for comparison. This geothermometer assumes chlorite + quartz to be in equilibrium and utilizes the activities of chlorite end-members.

**Table 7:** Variations of chlorite crystallization temperatures obtained using particular empirical geothermometers.

Chlorite type	Replacive chlorites (after pyroxene)		Non-replacive, colourless and radiating crystals associated with celadonite
	Massive, dark-green, granular and/or bundle-like aggregates	Fibrous, pale-green (locally porous) crystals	
Analytical points	14	17	16
Reference/element(s)-based:	Av. (*min.; max*); σ	Av. (*min; max*); σ	Av. (*min; max*); σ
Cathelineau^41^/Al^IV^	142 (124; 164); 16	156 (148; 166); 4	134 (122; 144); 6
Kavelieris et. al.^40^/Si	107 (106; 108); 1	107 (106; 108);1	107 (106; 109); 1
Jowett^44^/Al^IV^–(Fe/Fe + Mg)	148 (131; 170); 15	159 (151; 168); 4	131 (120; 141); 6
Zang and Fyfe^43^/Al^IV^–(Fe/Fe + Mg)	139 (128; 155); 11	158 (151; 165); 3	161 (152; 168); 5
El-Sharkawy^42^/Al^IV^–(Fe/Fe + Mg)	136 (125; 151); 10	150 (144; 157); 3	145 (137; 152); 5

Av.—average value (mean); min.—minimum value; max.—maximum value; σ—standard deviation.

**Figure 10:**
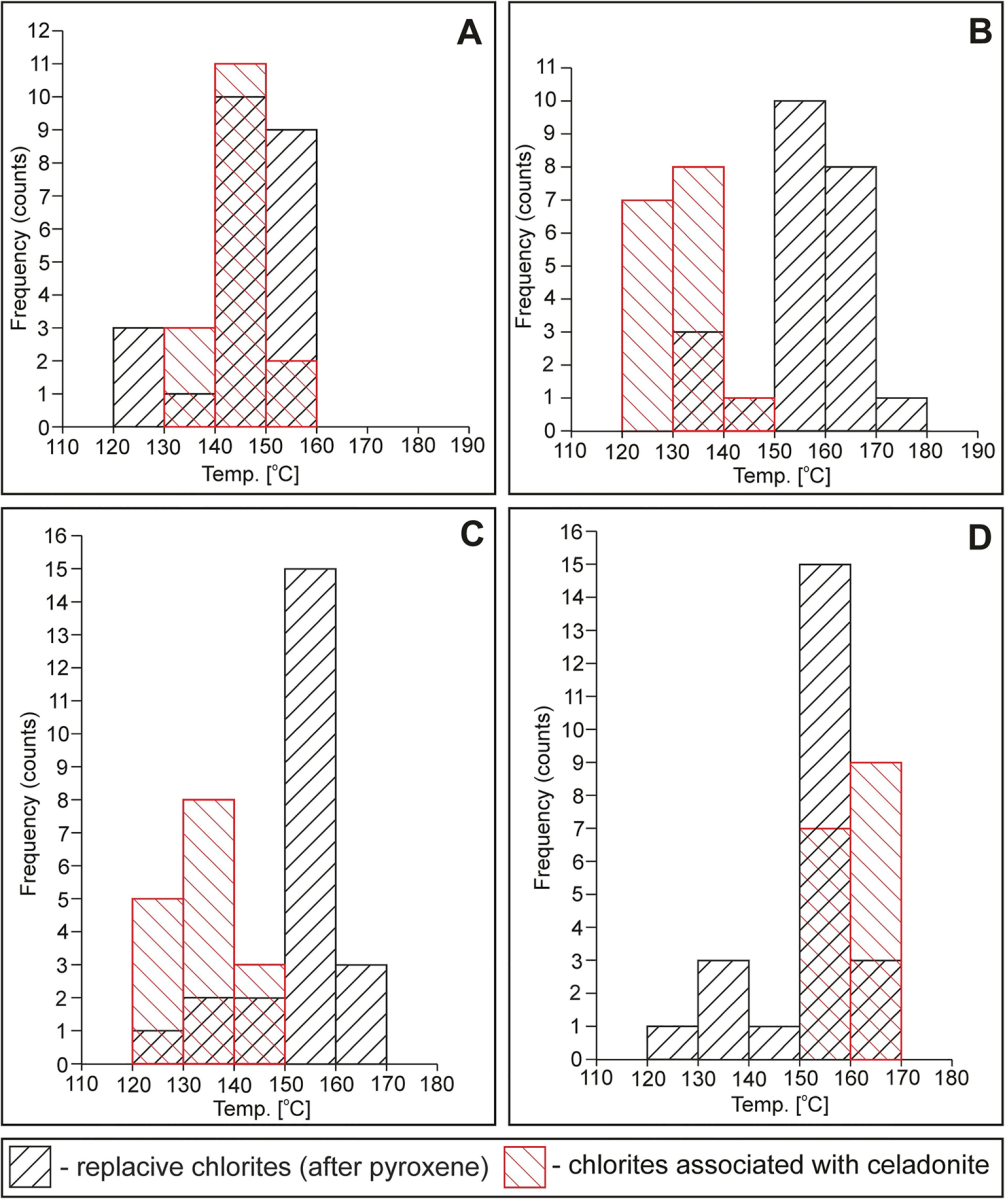
Frequency histograms showing calculated temperatures of replacive chlorites and chlorites associated with celadonite (non-replacive) based on the empirical (chemical) geothermometers: (**A**) Al^IV^- and Fe/(Fe + Mg)-based^42^, (**B**) Al^IV^- and Fe/(Fe + Mg)-based ^44^, (**C**) Al^IV^-based^41^, and (**D**) Al^IV^- and Fe/(Fe + Mg)-based^43^.

**Figure 11:**
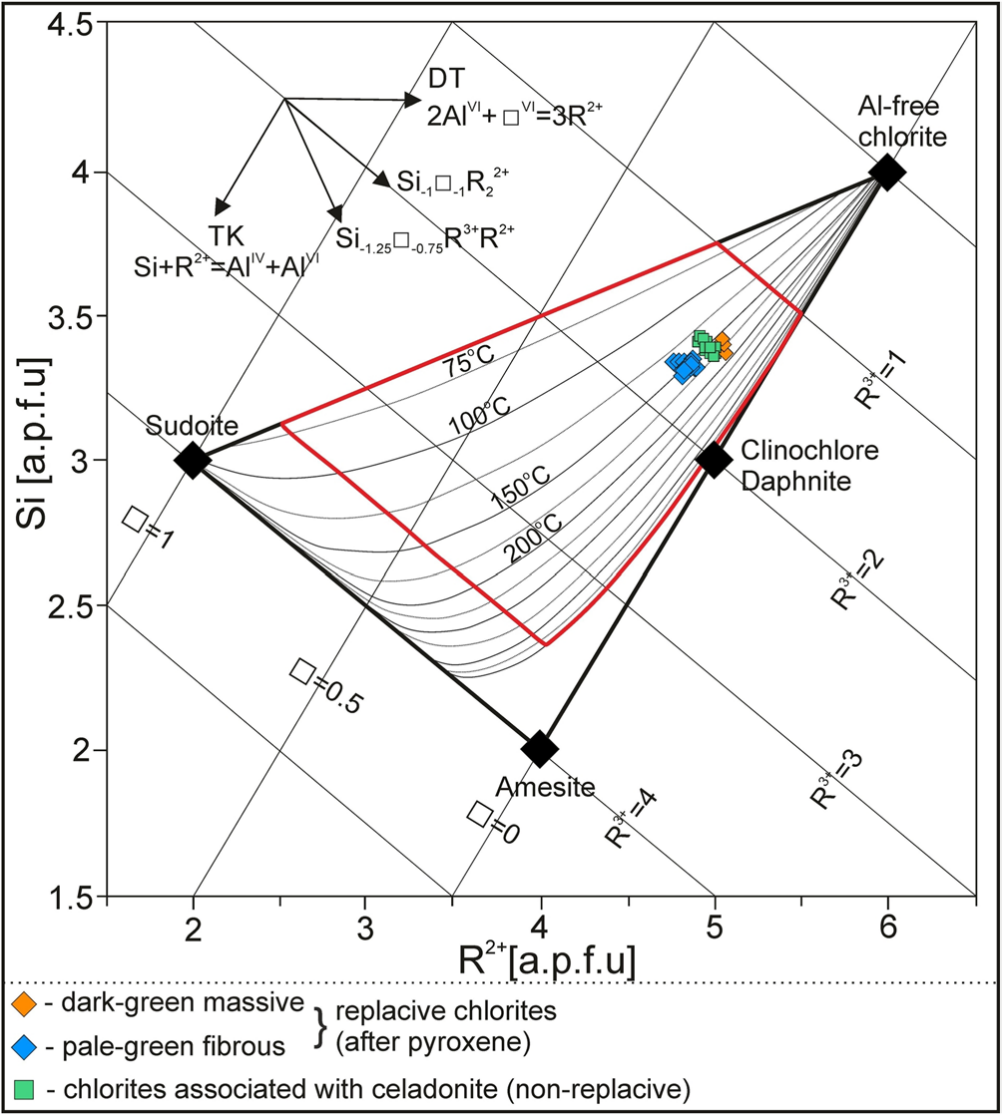
Distribution of temperatures obtained for replacive chlorites and chlorites associated with celadonite (non-replacive) within *T*–R^2+^–Si diagram^45^.

As a result, it was found that the formation temperature of replacive chlorites, i.e. both massive dark-green (1) and fibrous pale-green (2) varieties could be in the range of 106–170 °C (Table 7). Overall, the lowest temperature values (~ 107 °C) were provided according to the formula proposed by Kavalieris et al.^40^. The low formation temperatures of chlorites seem to be also supported by quite high octahedral vacancy (up to 0.40 a.p.f.u.), which tends to decrease along with increasing formation temperature^46^. The other geothermometers yielded fairly similar values, i.e. 124–166 °C^41^, 131–170 °C^44^, 128–165 °C^43^, and 125–157 °C^42^ for replacive chlorites of (1) and (2) type. Additionally, the local presence of high alkalis (Na + Ca + K) in dark-green massive replacive chlorites (1) is typical of low-temperature chlorites found in basaltic rocks and may account for i.e. the mixed-layer chlorite-smectite phases^47^. The distribution of formation temperatures is roughly unimodal as shown in Fig. 10. The results obtained from empirical (chemical) geothermometers are also consistent with *T*-R^2+^-Si plot^45^ as the analytical points mostly fall close to the 150 °C isotherm (Fig. 11) for the fibrous pale-green type of chlorite (2). Otherwise, massive dark-green chlorite (1) plots between 150 and 200 °C isotherm. Since the reliability of the semi-empirical geothermometer is partially maintained by the knowledge of Fe^2+/^Fe^3+^ ratios, these results possibly reflect the presence of some amounts of Fe^3+^ in chlorite structure and corresponding overestimation of R^2+^ (sum of divalent cations) parameter. The non-replacive chlorite (3) associated with celadonite is believed to crystallize at roughly similar temperature conditions (Table 7; Fig. 10). Its average formation temperatures were estimated in the range of 120–168 °C according to particular equations based on Al^IV^ content and/or Fe/Fe + Mg ratios. Following *T*–R^2+^–Si plot^45^, the formation temperature of non-replacive chlorites spanned the range of 125–175 °C (Fig. 11). Conversely, notably lower values (~ 107 °C) were revealed by Si-based geothermometer after Kavalieris et al. ^40^.

### Apatite fission-track dating (AFT)

**Figure 12:**
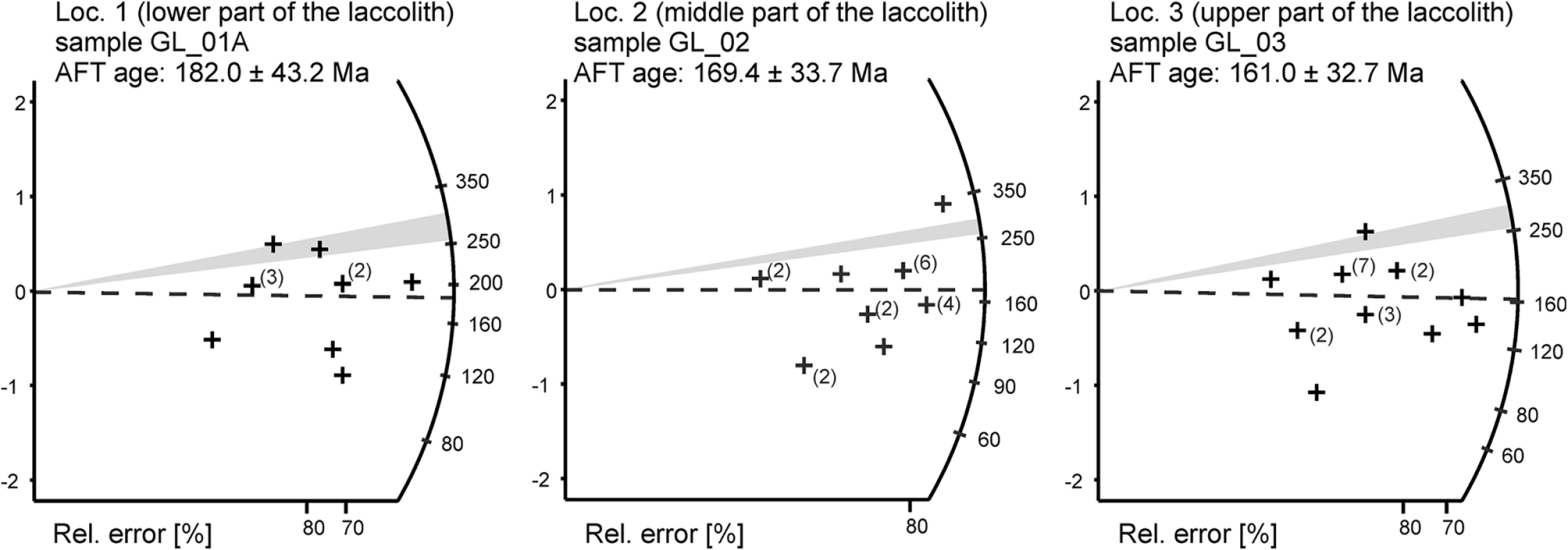
AFT radial plots of the samples from various areas of the laccolith from Głuszyca; Note that the inferred timing for magma emplacement is indicated by grey strips. The numbers in brackets indicate how many fluorapatite crystals yielded the same AFT age.

The obtained results of AFT analyses are presented in Tables 8 and 9. The samples have passed the chi-squared test (χ^2^) that confirms the homogeneity of the ages. Central ages obtained from AFT dating span from 182 ± 43.2 to 161.0 ± 32.7 Ma and thus roughly correspond to the Middle-Jurassic period (Fig. 12). Confidence intervals for single-grain ages are provided in Supplementary Data 2 and 3 for the full data set The halogen contents of fluorapatite crystals (F, Cl, and OH) were constant according to EMPA results (Table 5), and thus do not account for the variations in density of the spontaneous tracks ^48^. The total amount of U is low and constant, as it falls in the range of 3.65–3.15 ppm. The oldest ages (182.0 ± 43.2 Ma) were obtained for the sample GL_01A collected from lower areas of the magmatic body (Loc. 1—see Fig. 2A), which has been affected by the least extent of albitization and chloritization of primary plagioclase and pyroxenes, respectively. The results of AFT dating for the sample GL_02 from Loc. 2, marked by pervasive spilitization coupled with the abundance of porphyric microtextures, are slightly lower and yielded the central age of 169.4 ± 33.7 Ma. Similar AFT ages (161.0 ± 32.7 Ma) have been recorded from the rocks collected from the upper regions of the magmatic body (sample GL_03), which contain strongly albitized andesine-labradorite and show strong enrichment in iron oxides. The mentioned variations of central AFT data should be, however, treated with caution owing to low U content of the investigated fluorapatite and corresponding large errors (up to 43.2 Ma). Nonetheless, all AFT ages are younger than the igneous emplacement of the magmatic body, which occurred during the early-Permian period (ca. 290 Ma, although the exact age remains unknown due to lack of detailed geochronological studies and absence of i.e. zircon, which could be useful to obtain crystallization age).

**Table 8:** Apatite fission-track ages and sample details for the rocks collected from various areas of Głuszyca trachyandesite laccolith.

Position	Sample no	Alterations	Micro-textures	N_c_	Dosimeter		Spontaneous tracks		Induced tracks		U [ppm]	P (χ2) [%]	Central age [Ma] ± 1σ
					ρ_d_	N_d_	ρ_s_	N_s_	ρ_i_	N_i_			
Loc. 1	GL_01A	Partial albitization and chloritization	Fine-crystalline	11	1.19	3561	0.31	34	0.34	38	3.65	99.66	182.0 ± 43.2
Loc. 2	GL_02	Pervasive albitization, and chloritization, celadonitization	Porphyric	19	1.20	3592	0.26	47	0.31	57	3.26	100	169.4 ± 33.7
Loc. 3	GL_03	Pervasive albitization and hematitization	Fine-crystalline	20	1.19	3582	0.25	44	0.31	56	3.15	100	161.0 ± 32.7

Nc—number of crystals; *ρs,ρi,ρd*—density of spontaneous, induced and detector tracks, respectively (× 10^6^ tracks for cm^−2^); *Ns, Ni, Nd*—number of counted spontaneous, induced and detector tracks; P (χ^2^)—probability obtaining Chi-square value for n degree of freedom (where n = no. crystals-1^80^.

AFT data corresponds to central ages ± 1σ uncertainty. The external detector method and the ζ calibration approach was used to determine the fission tracks age^77,78^), with theζ values of 348.18 ± 6.52 for CN5 glass dosimeters (operator: A.A. Anczkiewicz).

Note the separation of apatite failed for sample GL_01B.

**Table 9:** Apatite fission-track length and Dpar (mean etch-figure diameter parallel to the *c* axis) data for the samples collected from various areas of the Głuszyca trachyandesite laccolith. Note the sample from Loc. 1 was excluded due to insufficient amounts of confined tracks.

Position	Sample no	Alterations	Micro-textures	nCT	CT mean [µm]	CT SD [µm]	CT skew	n Dpar	Dpar mean [μm]	Dpar SD [μm]	Dpar skew
Loc. 2	GL_02	Pervasive albitization, and chloritization, celadonitization	Porphyric	52	12.23 ± 0.26	1.90	0.03	41	2.12	0.36	− 0.48
Loc. 3	GL_03	Pervasive albitization and hematitization	Fine-crystalline	27	12.40 ± 0.29	1.49	− 0.33	39	2.00	0.37	0.39

nCT—number of measured confined tracks; CT mean—mean confined track length; SD—standard deviation; CT skew—skewness of distribution relative to the mean value (measure of asymmetry of the distribution); n Dpar—number of etch pit diameters measured; Dpar mean: mean etch pit diameter; Dpar SD—standard deviation of etch pit diameters; Dpar skew: skewness of etch pits.

**Figure 13:**
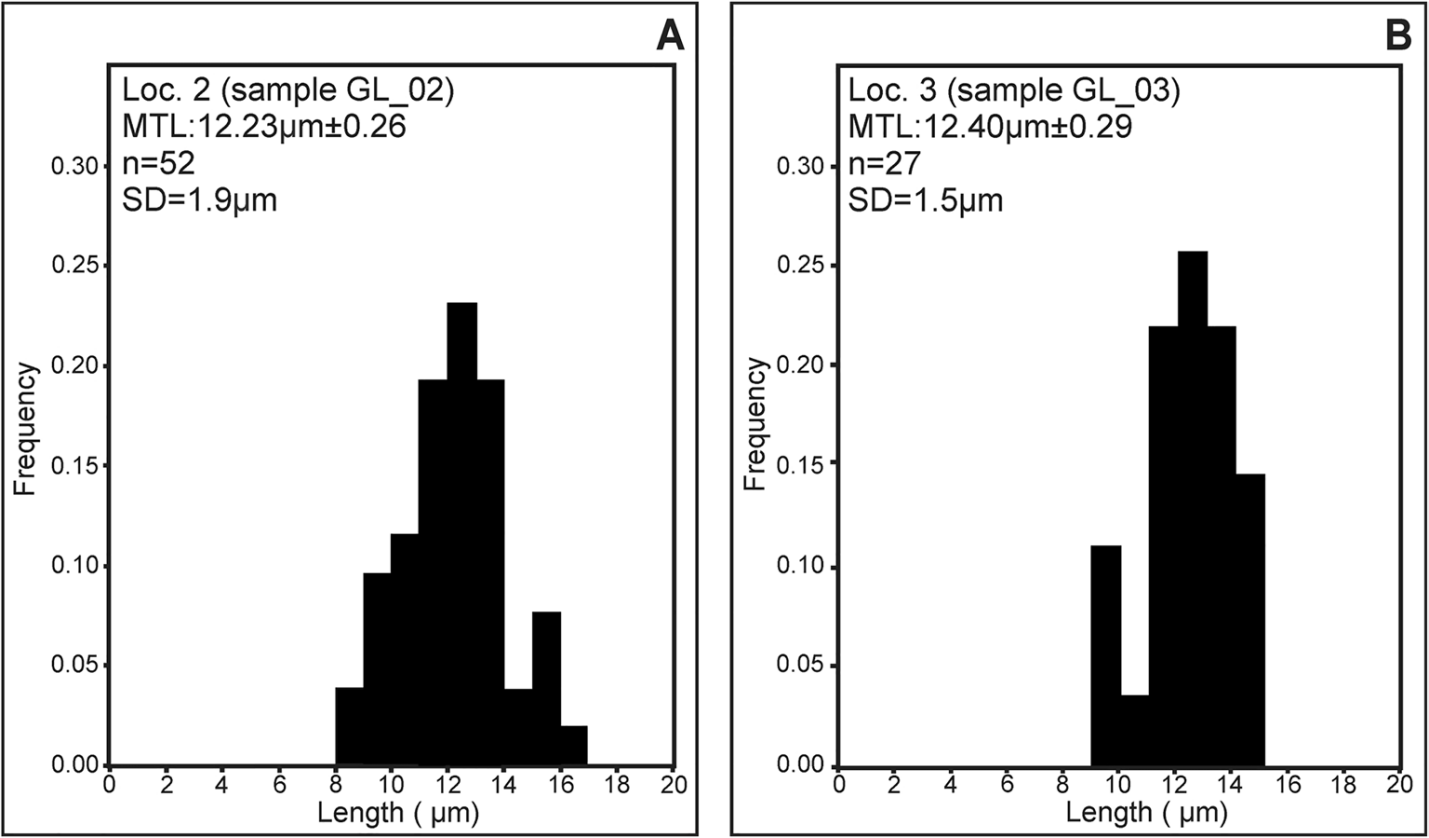
Apatite fission-track length data for the samples from Loc. 2 (middle part of the laccolith) (**A**) and 3 (upper part of the laccolith) (**B**). Mean track lengths (MTL), number of measured confined tracks (n), and standard deviations (SD) were included within particular histograms. Note the sample from Loc. 1 (lower part of the laccolith; GL_01A) was excluded due to the insufficient amounts of confined tracks.

Confined track lengths were measured for the samples GL_02 and GL_03 from Loc. 2 and 3 (n = 52 and n = 27, respectively)—see Table 9. The confined track-length distribution of the spontaneous tracks could be described as fairly unimodal (Fig. 13), with mean measured confined tracks lengths (MTL) of 12.23 ± 0.26 μm (GL_02) and 12.40 ± 0.29 μm (GL_03). Additionally, the standard deviation (SD) of MTL is low for grains found in both samples (GL_02—1.90 μm; GL_03—1.49 μm). Mean Dpar values, found in the range of 2.0–2.12, are typical of fluorapatite, which is marked by a relatively high susceptibility to annealing ^48^.

### Whole rock major and trace element data

The samples from various areas of the laccolith are characterized by constant and intermediate SiO_2_ contents in the range of 54.12–59.80 wt.%, as well as fairly similar Al_2_O_3_ concentrations (15.50–17.45 wt.%)—Table 10. They reveal either metaluminous (sample GL_01A) or peraluminous (other samples; i.e. GL_01B, GL_02, and GL_03) nature according to the values of A/NK (molar Al_2_O_3_/Na_2_O + K_2_O ratio) and A/CNK (molar Al_2_O_3_/CaO + Na_2_O + K_2_O ratio) in the overall range of 1.27–1.50 and 0.95–1.16, respectively. The contents of FeO_T_ occur in the narrow range of 6.06–7.73 wt.%, whereas MgO is quite variable and falls between 1.83 and 4.40 wt.%. CaO contents (1.56–2.93 wt.%) are generally low as compared with Na_2_O (2.31–5.89 wt.%) and K_2_O (2.58–7.25 wt.%). Meanwhile, the highest concentrations of Na_2_O (up to 5.89 wt.%) has been reported in samples from lower parts of the laccolith where the presence of sodium pyroxenes (aegirine) has been reported. The concentrations of TiO_2_, P_2_O_5_, and MnO (1.05–1.41; 0.42–0.61; and 0.06–0.08 wt.%, respectively) are low and do not show remarkable difference among the samples. The rocks are also poor in such transition elements as Cr, Ni, and Co. Lost on ignition (L.O.I.) values exhibit moderate variations and range from 2.2 wt.% in samples from lower parts of the laccolith (i.e. GL_01A) to 4.30 wt.% in sample GL_03 (upper part of the laccolith) owing to the variable alteration degree, as well as the presence of calcite.

**Table 10:** Whole rock major and trace element data for the samples collected from various levels of Głuszyca trachyandesite laccolith.

Locality	Loc.1 (lower parts of the laccolith)				Loc.2 (middle parts of the laccolith)		Loc.3 (upper parts of the laccolith)
Sample type	GL_01A	GL_01A	GL_01B	GL_01B	GL_02	GL_02	GL_03
**Major element (wt.%)**							
SiO_2_	59.68	59.8	57.2	56.65	57.75	57.57	54.12
TiO_2_	1.05	1.06	1.39	1.41	1.30	1.34	1.58
Al_2_O_3_	16.80	16.69	15.5	15.66	16.17	16.27	17.45
FeO_T_	6.06	6.14	7.64	7.73	7.13	7.16	6.45
MnO	0.08	0.08	0.06	0.06	0.08	0.08	0.04
MgO	1.83	1.84	4.34	4.40	3.90	3.90	3.65
CaO	2.50	2.51	2.90	2.93	1.57	1.56	1.88
Na_2_O	5.89	5.81	4.03	4.00	5.34	5.47	2.31
K_2_O	3.27	3.23	2.58	2.61	2.61	2.60	7.25
P_2_O_5_	0.42	0.43	0.58	0.61	0.53	0.54	0.67
L.O.I	2.20	2.20	3.40	3.60	3.30	3.20	4.30
Total	99.81	99.81	99.74	99.72	99.76	99.74	99.77
Mg#	0.35	0.35	0.50	0.50	0.49	0.49	0.50
CaO/Na_2_O	0.42	0.43	0.72	0.73	0.29	0.29	0.81
A/NK	1.27	1.28	1.65	1.67	1.39	1.38	1.50
A/CNK	0.95	0.95	1.05	1.06	1.12	1.11	1.16
**Trace element (ppm)**							
Be	3	2	2	2	3	8	3
Sc	12	12	17	17	15	15	23
V	84	90	111	122	99	109	138
Cr	34.21	34.21	41.06	27.37	27.37	41.06	34.21
Co	13	13.4	17.6	18.4	15.8	15.8	7.3
Ni	b.d	b.d	b.d	b.d	b.d	b.d	b.d
Ga	16.2	17.8	18.4	19.2	16.2	17.5	20.6
Rb	65.8	71.8	62.2	65.8	64.5	67	127
Sr	280.4	286.4	135.5	138.7	98.4	100	24.8
Cs	0.3	0.3	1.2	0.9	1.1	1.3	2.5
Y	37.1	38.9	49	47.9	48.8	47.6	50.2
Zr	400.8	416.7	484.4	502.8	489.9	500.4	458.1
Nb	26.8	27.7	34.7	35.6	34.5	34.9	43.4
Ba	524	523	655	663	564	541	274
Hf	8.9	9.2	10.9	11.1	11.4	11.1	12.2
Ta	1.5	1.5	2.2	2	2.1	1.8	2.1
Th	9.9	10.6	11.2	11.8	11.9	12.2	14.4
U	2.1	2.4	5.4	5.7	4.6	4.9	5.5
La	57.5	62.5	75.6	81.5	79.1	82.5	85.3
Ce	112	117.4	148.5	157.6	152.9	155.7	167.5
Pr	13.56	13.74	18.32	18.31	18.57	18.12	20.61
Nd	50.8	52.8	67.2	68.1	67.6	67.1	77.2
Sm	9.06	8.87	12.42	12.31	12.34	11.87	13.79
Eu	2.11	2.07	2.7	2.65	2.67	2.63	2.71
Gd	8.1	8.1	10.78	11.84	10.69	10.67	11.86
Tb	1.21	1.22	1.59	1.6	1.57	1.59	1.71
Dy	7.08	6.79	8.99	8.83	8.76	8.74	9.73
Ho	1.37	1.41	1.77	1.76	1.79	1.71	1.92
Er	3.89	3.78	4.87	4.92	5.12	5.06	5.65
Tm	0.55	0.56	0.67	0.7	0.72	0.71	0.8
Yb	3.4	3.5	4.47	4.5	4.61	4.56	5.24
Lu	0.51	0.52	0.68	0.7	0.72	0.72	0.8
REE sum	271.14	283.26	358.56	375.32	367.16	371.68	404.82
[La/Lu]_CN_	11.70	12.48	11.54	12.08	11.40	11.89	11.07
[La/Sm]_CN_	3.96	4.40	3.80	4.13	4.00	4.34	3.86
[Gd/Lu]_CN_	1.96	1.93	1.96	2.09	1.84	1.83	1.83
Eu/Eu*	0.75	0.74	0.71	0.67	0.71	0.71	0.65

L.O.I.—loss on ignition; b.d.—below detection limit; Mg#— molar MgO/(MgO + FeO); Eu* = (Sm·Gd)^1/2^; _CN_—chondrite-normalized; _PM_—primitive-mantle normalized. A/CNK = molar [Al_2_O_3_/(CaO + Na_2_O + K_2_O)]; A/NK = molar [Al_2_O_3_/(Na_2_O + K_2_O)].

**Figure 14:**
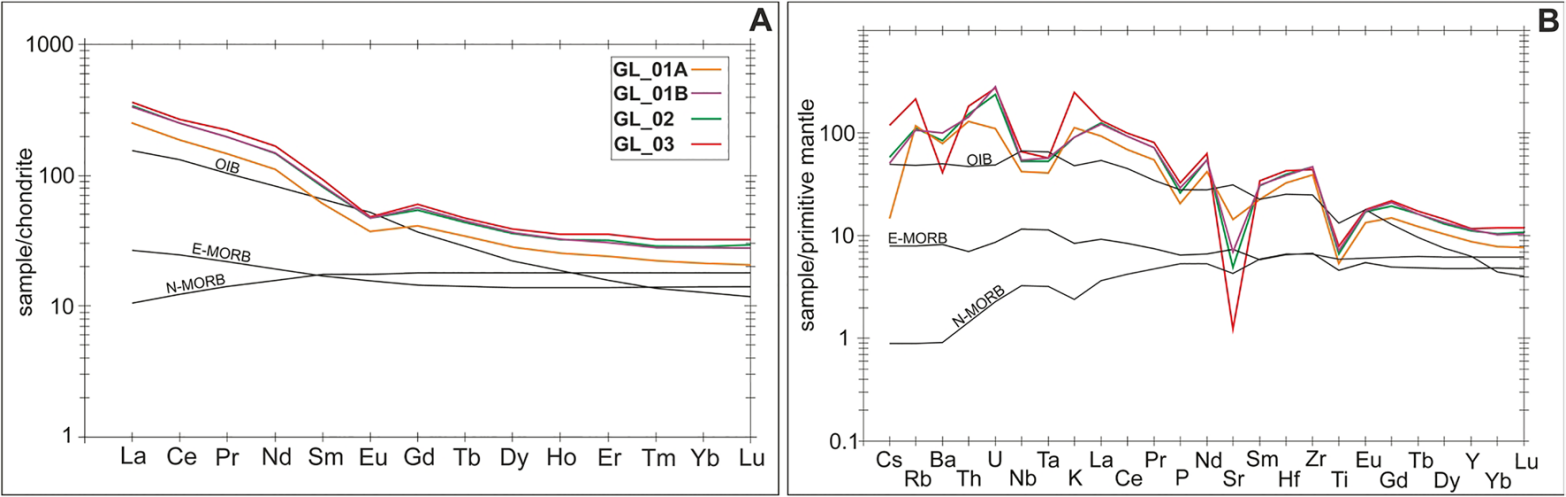
(**A**) Chondrite-normalized REE patterns and (**B**) primitive mantle-normalized multi-element diagrams for trachyandesites from Głuszyca (note the curves for samples GL_1A, GL_01B, and GL_02 represent average composition of two analyses per sample**—**see Table 10). Normalization values are after ^84^. The curves for OIB, N-MORB, and E-MORB basalts were included. For detailed description of particular samples and their alteration style, the reader is referred to Table 1.

The chondrite-normalized rare-earth element (REE) patterns for the samples are coherent and reveal rightward-inclined shape owing to the enrichment of light rare-earth elements (LREE; La-Sm) relative to heavy rare-earth elements (HREE; Gd-Lu), as evidenced by i.e. high [La/Lu]_CN_ ratios (11.07–12.48)—Fig. 14A. The curves for LREE display relatively strong fractionation due to the elevated [La/Sm]_CN_ ratios in the range of 3.80–4.40. Conversely, HREE segments are quite flat with [Gd/Lu]_CN_ ratios between 1.83–2.09. All samples are marked by the presence of subordinate Eu anomaly as [Eu/Eu*] ratios fall in the range of 0.65–0.75. The primitive-mantle normalized multi-element spider diagrams for the samples are also fairly parallel to each other and consistent in terms of high-field strength elements HFSE (i.e. Zr, Nb, Ta) and REE abundances—Fig. 14B. The curves are characterized by negative troughs for Nb and Ta and corresponding enrichment in LREE, as well as in U and Th. Otherwise, LILE (large ion lithophile elements) such as Cs, Rb, Ba, Sr, and K show remarkable variations in the mantle-normalized patterns. The strongly-altered samples from upper parts of the laccolith (i.e. GL_03) are relatively depleted in Sr and Ba, but enriched in Cs, Rb, and K relative to the samples from lower parts of the laccolith (with lower alteration degree—i.e. GL_01A-B). Additionally, the sample GL-01A from the lowermost part of the laccolith shows notably lower U contents. All samples are marked by slightly positive Zr and Hf spikes relative to HREE, coupled with notable negative anomalies for Ti and P.

## Discussion

### The origin and nature of spilitic assemblages

The formation of spilitic assemblages in Głuszyca trachyandesite laccolith involved chloritization of primary pyroxenes (i.e. augite) and albitization of primary plagioclases (andesine-labradorite). These alterations were followed by the concomitant formation of celadonite and vug-filling chlorites, as well as iron oxides (after i.e. primary magnetite/ilmenite) in the uppermost parts of the laccolith. It is also noteworthy, that the abundance of calcite indicates high activity of CO_2_ during metasomatic processes, whereas the formation of secondary titanite could be triggered by the release of Ca^2+^ and Ti^4+^ during the breakdown of pyroxene and magnetite/ilmenite, following such reaction as:

$$\left( {\text{I}} \right) \, \;\;\;\;\;{\text{Ca}}^{{{2} + }}_{{({\text{augite}})}} + {\text{Ti}}^{{{4} + }}_{{({\text{ilmenite}})}} + {\text{ SiO}}_{{{2}({\text{fluid}})}} + {\text{3H}}_{{2}} {\text{O}} \to {\text{CaTiO}}_{{5}} \left( {{\text{titanite}}} \right) + {\text{6H}}^{ + }$$

Although spilitic alterations have strongly overprinted the original mineralogical features of the trachyandesites, the samples from lower parts of the laccoliths still provide some information on primary magmatic assemblages. Particularly, these samples contain unique association of sodium- and calcium-bearing pyroxenes (i.e. aegirine mantled by augite—see Fig. 4B). Although augite is quite common in volcanogenic rocks from the ISB basin where it has been reported from poorly-spilitized samples^21^, the occurrence of aegirine has not been reported elsewhere in that study area. However, it remains ambiguous whether aegirine represents primary (I) or secondary (II) phase hosted by volcanics from Głuszyca trachyandesites. Firstly, direct crystallization from magma (I) is not supported by geochemical (meta- to peraluminous) character of the samples and associated mineral assemblages. Conversely, (II) secondary replacive or even authigenic low-temperature (see i.e.^49^) nature does not conform to microtextural observation, i.e. the presence of augite overgrowing on aegirine, as well as the possible chloritization of aegirine. Moreover, aegirine occurs as relatively large prismatic crystal found exclusively in less altered rocks; no sodic pyroxene occurs in any strongly-albitized samples. Thus, its presence cannot be explicitly related to the formation of other Na-bearing minerals (i.e. secondary albite) as a result of Na metasomatism. Alternatively, aegirine crystals may be interpreted as xenoliths, although further investigations are necessary to support such a type of scenario.

The formation of secondary albite is common in spilitic rocks worldwide and has been widely reported in volcanic rocks from the ISB, including the samples investigated in the following study. The mineralogical and microtextural characteristics of secondary replacive albite, characterized by weak to dark-brown cathodoluminescence and almost pure chemical composition with Ab ~ 99 mol.%, gives a clue for its formation conditions in the Głuszyca trachyandesites. These features are typical of authigenic and/or diagenetically-altered feldspars (i.e.^50^) and correspond to the ordering of crystal lattice and/or vanishing of structural defects within secondary albite during low-temperature fluid-mineral interactions (i.e.^51^). The vanishing CL intensity within secondary albite might be also related to the incorporation of structural water (i.e. OH^-^ groups)^52^. Meanwhile, the albitization is mainly developed in the interior of primary plagioclase laths (andesine-labradorite), and thus was likely enhanced by i.e. the presence of polysynthetic twinning in primary andesine-labradorite or cleavage planes and other internal fractures (cf.^1^). Based on EMPA and microtextural data, the transition from primary plagioclase to secondary albite could follow simplified, allochemical, and Al-conservative reaction:

$$\left( {{\text{II}}} \right)\;\;\;\;{\text{Na}}_{{0.{5}}} {\text{Ca}}_{{0.{5}}} {\text{Al}}_{{{1}.{5}}} {\text{Si}}_{{{2}.{5}}} {\text{O}}_{{8}} \left( {{\text{andesine}}} \right) \, + {\text{ Na}}^{ + } \left( {aq.} \right) \, + {\text{ 2H}}_{{4}} {\text{SiO}}_{{4}} \left( {aq.} \right) \, \to { 1}.{\text{5NaAlSi}}_{{3}} {\text{O}}_{{8}} \left( {{\text{albite}}} \right) \, + 0.{\text{5Ca}}^{{{2} + }} \left( {aq.} \right) + {\text{4H}}_{{2}} {\text{O}}$$

Alternatively, albitization could also reflect the constant-volume reaction that assumes some quantities of Al^3+^ to be released during mineral replacement reaction:

$$\left( {{\text{III}}} \right){\text{ Na}}_{{0.{5}}} {\text{Ca}}_{{0.{5}}} {\text{Al}}_{{{1}.{5}}} {\text{Si}}_{{{2}.{5}}} {\text{O}}_{{8}} \left( {{\text{andesine}}/{\text{labradorite}}} \right) \, + \, 0.{\text{5Na}}^{ + } \left( {aq.} \right) + \, 0.{\text{5H}}_{{4}} {\text{SiO}}_{{4}} \left( {aq.} \right) \, \to {\text{ NaAlSi}}_{{3}} {\text{O}}_{{8}} \left( {{\text{albite}}} \right) + \, 0.{\text{5Ca}}^{{{2} + }} \left( {aq.} \right) + \, 0.{\text{5Al}}^{{{3} + }} + {\text{ 2OH}}^{ - }$$

The reaction (2) seems to be more relevant in this case as the secondary albite in Głuszyca trachyandesites is devoid of Al-bearing phases such as mica and/or epidote unless they are represented by nano-scale inclusions, which cannot be visible under BSE images. Such phases have been, however, frequently recognized as cogenetic inclusion found within secondary albite from albitized granitic rocks (e.g. ^2^).

The continental character of Głuszyca trachynadesites (and other volcanics from the ISB) precludes the role of magma-water interactions during the formation of secondary albite typical of spilitic rocks. Thus, magmatic-related Na-rich fluids could serve as a source of Na^+^ and Si^4+^ (cf. ^15^), both elements necessary for albitization of andesine-labradorite (see reactions II and III). The development and extent of albitization might be possibly related to such factors as: the geochemical character of trachynadesite-forming magma (i.e. enrichment in alkalis), high activity of CO_2_, sodium-rich character of primary plagioclase (i.e. andesine) that require relatively less amounts of Na^+^ in alternating fluids, and/or subvolcanic rather than *stricte* extrusive character of volcanic rocks from the ISB. Nevertheless, it is also possible that some amount of Na^+^ could be derived from chloritization of Na-bearing pyroxene (aegirine) since replacive Mg-chlorite represents Na-free species that could incorporate only such elements as Al, Si, and Fe during the breakdown of primary pyroxene. Thus, Na released during the breakdown of aegirine could be further fixed with secondary albite.

### Constraints on the timing of spilitization

The fluorapatite separated for AFT dating represents a late-magmatic phase as evidenced by its size variations (from several µm up to ca. 0.4 mm), coupled with the occurrence of hollow-type tubes and swallow-type (H-shaped) terminations within particular crystals, visible during both OM-CL and SEM-BSE observations. These fabrics were generally reported from minerals that have been subjected to quenching (rapid cooling) and may reflect rapidly-increased growth rate during magma eruption^53^.

According to Gleadow et al.^54^, AFT dating of magmatic rocks does not always indicate the age of their igneous emplacement and/or early-stage crystallization, since the closure temperature of apatite falls within the range 70–110 °C (so-called Apatite Partial Annealing Zone; APAZ). The absolute (“magmatic”) ages may be, however, obtained in specific cases, i.e. assuming that particular rocks cooled quickly, being remained close to the surface, and were subsequently unaffected by any remarkable thermal disturbances. The obtained AFT results (161–182 Ma) are significantly younger than the emplacement of the magmatic body, which occurred during Middle Rotliegendes (~ 299–271 Ma) and should be considered as apparent ages that do not reflect the timing of apatite crystallization, but were possibly overprinted by a younger thermal event. These ages cannot be explained by i.e. prolonged residence above APAZ, burial metamorphism, and/or compositional variations of apatite (i.e. variable Cl content). Meanwhile, several lines of evidence indicate that AFT results (161–182 Ma) can indicate the timing of ubiquitous alterations (spilitization), including chloritization of pyroxenes (augite and aegirine) combined with albitization of primary plagioclases (andesine-labradorite). Firstly, (1) the crystallization temperatures obtained from chlorite thermometry (106–170 °C in the case of both replacive and vug-filling chlorites) indicate that alternating fluids were capable of resetting the AFT system, since APAZ falls into ca. 70–110 °C. (2) Secondly, the AFT ages (161–182 Ma) are quite consistent with the K–Ar dating results of amygdule-filling celadonite (177–252 Ma), which has been encountered among early-Permian continental volcanics from the closed Lubiechowa quarry in the North-Sudetic Basin^55^. (3) Finally, similar (Middle Jurassic) intercept age has been recently constrained using U–Pb dating of hydrothermal euhedral anatase, found in Carboniferous clastic deposits from the Intra-Sudetic Basin^56^.

Relatively short mean confined track lengths (12.23–12.40 µm), coupled with uniform distribution of track lengths and low values of standard deviations (1.5–1.9 µm), suggest almost total reheating of the rocks due to invasion of metasomatizing spilitization-related fluids. The variations of AFT ages obtained from various areas of the magmatic body (from 182 to 161 Ma) possibly account for the variable progress of alteration processes and /or differences in cooling rates.

### Geodynamic implications

AFT ages of the trachyandesites from Głuszyca not only reflect the timing of spilitization, but are also significantly younger as compared with the current AFT data of both crystalline basement and magmatic-sedimentary succession in the Intra-Sudetic Basin^28,29^. Overall, two major large-scale geological events have strongly imprinted the low-temperature cooling history of the Intra-Sudetic Basin infill: (1) burial under Mesozoic sediments, followed by Europe-Africa-Iberia plate convergence (85–70 Ma) that triggered the inversion of the Intra-Sudetic Basin, and subsequent reactivation of faults and thrusts^57,58^ as well as (2) Paleogene-Neogene reheating associated with the widespread magmatic activity, which has been triggered by the opening of Eger rift^59^. Therefore, the AFT results of both magmatic and sedimentary rocks found within the Intra-Sudetic Basin are often ambiguous and may lead to various tectonomagmatic implications (cf.^27,60^). Meanwhile, there are still some contradictions whether the partial reset of AFT ages in the Intra-Sudetic Basin was linked to the transgression of a shallow-level sea during the Cenomanian. This transgression resulted in the deposition of large piles of sediments up to ca. 900 m in thickness that could be responsible for complete and/or partial reheating of sedimentary and/or magmatic rocks above apatite partial annealing zone (70–110 °C). According to Danišík et al.^60^ and Sobczyk et al.^28^, and Botor et al.^29^, the sediments related to the Cenomanian transgression had covered almost the entire area of the Intra-Sudetic Basin and resulted in the pervasive reset of AFT ages for older deposits of Carboniferous and/or Permian age. Otherwise, such authors as i.e. Skoček and Valečka^61^, Biernacka and Józefiak^62^, and Uličný^63^ postulated the theory of “Western and Eastern Sudetic Islands”. According to this model, the Sudetes area could be rather exposed as an archipelago of islands (paleo-highs) emerging above the sea level and hence burial during the Cenomanian transgression had not necessarily affected some older deposits. However, to the best of our knowledge, the exact paleographic reconstruction of exact position, as well as extent of these island is still quite vague and ambiguous. The results of AFT dating obtained for trachyandesites from Głuszyca (161–182 Ma) indicate that these rocks have not preserved the record of large-scale geological events in the Intra-Sudetic Basin such as tectonic inversion and exhumation. These ages are also notably older than the transgression of the Cenomanian sea (~ 95 Ma), and hence seem to support the “Island scenario” in the Intra-Sudetic Basin, indicating no significant influence of Late-Mesozoic sediment deposition on the cooling history of the volcanic rocks. Thus, further investigations are necessary to eventually re-evaluate the low-temperature history of the ISB that involves the presence of emerged landmasses (“Sudetic Islands”) during the Cenomanian.

### Magma evolution and mobility of trace elements during low-temperature alterations

**Figure 15:**
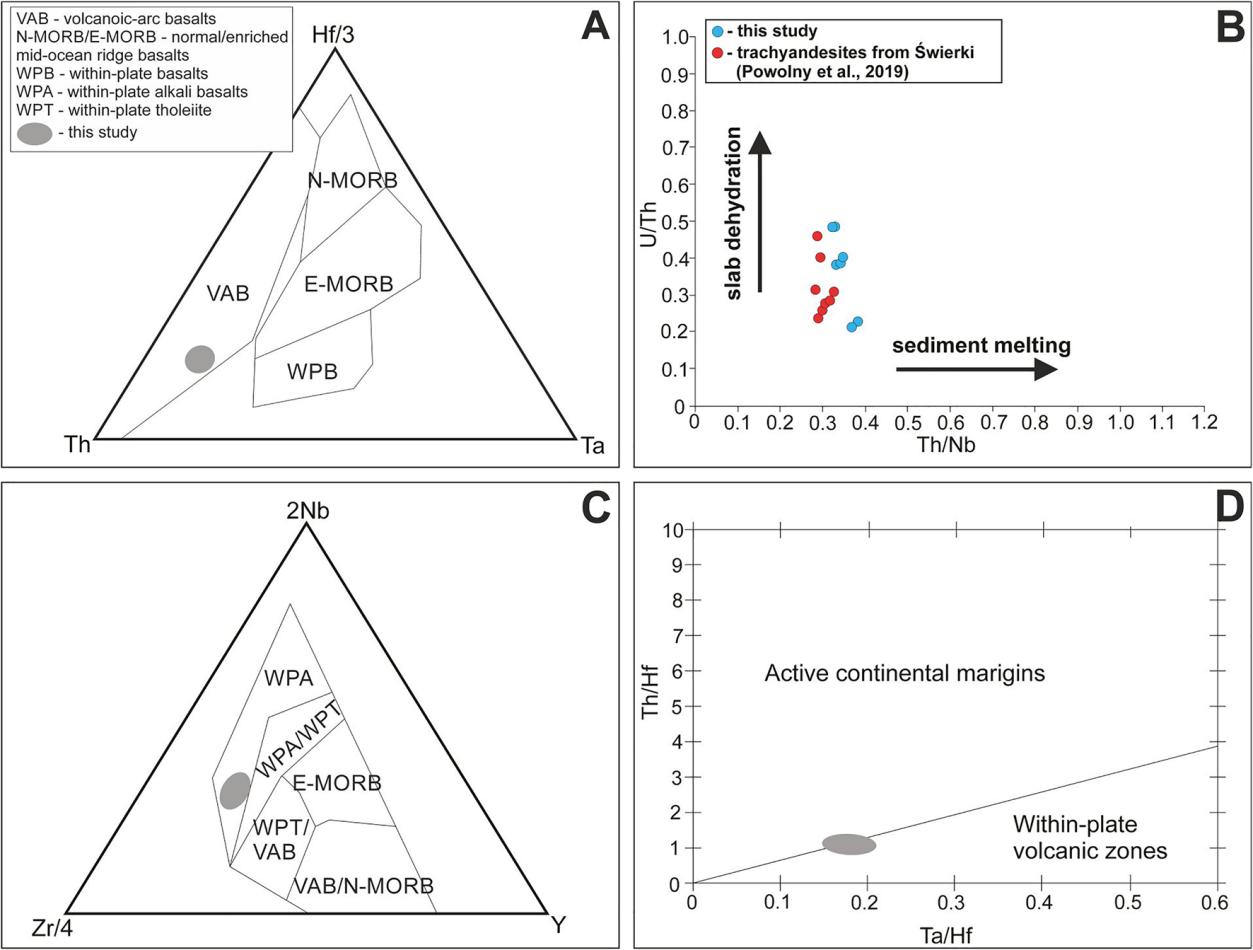
Examples of tectono-magmatic discrimination diagrams for trachyandesites from Głuszyca. (**A**) Th–Ta–Hf/3 discrimination plot^70^ showing volcanic-arc basalts affinity (VAB); (**B**) U/Th versus Th/Nb plot^73^, where the samples follow slab dehydration rather than sediment melting trend (both mechanisms can account for the presence of subduction-related signatures); note the position of trachyandesites from Świerki exposure in the ISB (ca. 3 km SW of Głuszyca exposure) has also been included in order to better visualize particular trends^21^; (**C**) 2Nb–Zr/4–Y discrimination plot^71^ showing within-plate affinity (WPA); (**D**) Ta/Hf–Th/Hf diagram^72^ where the samples straddle the boundary between arc- and within plate-related fields.

The samples have probably undergone some fractional crystallization prior to their emplacement, as shown by i.e. low contents of transition elements (e.g. Cr and Ni, Sc, V), and low MgO (< 4.5 wt.%), and low Mg# (0.35–0.50), that resulted from an early-stage separation of mafic minerals such as olivine. Negative Eu anomaly in chondrite-normalized patterns (Fig. 14A) can be ascribed to the fractionation of primary (Ca-bearing) plagioclase, whereas negative Ti and P anomalies in the mantle-normalized diagrams (Fig. 14B) may be at least partially linked to fractionation of titanite and apatite, respectively. LREE-enriched rare-earth patterns for the samples are typical of continental basalts^64^. Whereas, high LREE/Nb (i.e. La/Nb ratios of ca. 2.2) and LREE/Ta (i.e. La/Ta ratios of ca. 39.9), along with the corresponding Nb–Ta troughs in the mantle-normalized patterns (Fig. 14B) suggest the involvement of subduction-modified mantle source in the petrogenesis of the trachyandesites from Głuszyca. Nb/Th ratios found within the range of 2.6–3.1 are also typical of arc-related environments^65^. The hydrous melting is also evidenced by the occurrence of sparse amounts of amphibole group species within rock matrix. Otherwise, Nb/U ratios (6.3–12.8) are quite similar to those reported for continental crust (6–10; see:^66^). Low [Ta/U]_PM_ (< 0.4), enrichment in Zr (up to 502.8 ppm) and Hf (up to 12.2 ppm), as well as a bit humped-shaped mantle-normalized diagrams (Fig. 14B) also indicate crustal affinities (see e.g.^67,68^). Hence, it may be inferred that crustal contamination could have been strongly involved during the magma evolution. This conclusion is further supported by the presence of interstitial magmatic quartz within rock matrix. Moreover, the occurrence of pink-luminescent apatite (REE^3+^-activated), followed by yellow-luminescent (REE^3+^- and Mn^2+^-activated) outermost domains in particular crystals, indicate changes in melt chemistry (i.e. alkaline-acidic) during magma evolution^69^. The presence of mixed subduction-related and crustal affinities is further supported by various tectonic-setting discrimination diagrams, where the samples show either arc-related (due to i.e. modification of the source via subduction-derived components such as fluids) or within-plate affinity^70–73^—see Fig. 15A–D.

The mobility of trace elements in basaltic rocks during low-grade metamorphism such as spilitization is quite ambiguous. According to Herrmann et al.^74^, the trace element concentration of REEs and HFSEs is resistant to low-temperature alterations and remains constant in both weakly- and strongly-altered rock samples. Conversely, Hellman et al. (1979)^18^ suggested these elements are prone to i.e. spilitization, and thus cannot be properly used to i.e. discriminate tectonic setting of altered basaltic rocks. REE patterns for the trachyandesites from Głuszyca are consistent and seem to preserve the original LREE/HREE ratios in both weakly- and strongly-altered samples (cf. Fig. 14A). The mantle-normalized diagrams are also sub-parallel, but there are also some discrepancies in terms of some LILE (i.e. Cs and Sr), as well as U contents in samples of variable alteration degree—Fig. 14B. Firstly, the samples containing pyroxene relicts and partially-albitized plagioclase (i.e. sample GL_01A, Loc. 1, lower parts of the laccolith) are relatively rich in Sr, but depleted in Cs relative to the samples from upper parts of the laccolith that show increasing alteration degree (i.e. albitization and chloritization). These changes could be explained by leaching of Sr and Ba during plagioclase (and partially K-feldspar) alteration (i.e. albitization), whereas the enrichment is Cs could be possibly explained by the weathering of feldspars followed by the formation of hydrous phases (e.g. sericite). Depletion in Ba within the sample from upper parts of the laccolith could be attributed to either alteration/weathering or fractional crystallization of K-feldspars. Finally, variations in U contents (depleted in samples from lowermost parts of the laccolith) is linked to the variable amounts of apatite rather that secondary alterations effect, as evidenced by well-developed positive correlation between P_2_O_5_ and U (R^2^ = 0.88).

## Conclusions

1. Trachyandesites from Głuszyca laccolith-type magmatic body (Lower Silesia, the Intra-Sudetic Basin, SW Poland) contain spilitic assemblages including Mg-chlorite (after augite and/or aegirine), albite (after andesine-labradorite), as well as secondary titanite, calcite, celadonite, and vug-filling chlorite. Sodium input could be either triggered by magmatic-related CO_2_-rich, Na- and Si-rich fluids, although Na^+^ and Si^4+^ could also originate from the early breakdown of sodium-bearing pyroxene (aegirine), which has been recognized for the first time in volcanogenic rocks from the Intra-Sudetic Basin.
2. Albitization was crystallographically-controlled mechanism that resulted in the formation of weakly luminescent to dark-brown luminescent, almost pure (Ab > 99 mol.%) secondary albite forming elongated patches developed within interior of the host andesine-labradorite (~ An_47_Ab_50_Or_3_). These features are typical of diagenetically-altered (low-temperature) feldspars and support rather low-temperature character of fluid-rock interactions during late or post magmatic stage. Moreover, Al^3+^ released during albitization of andesine-labradorite could be entirely consumed by secondary albite and thus cogenetic Al-bearing phases (i.e. epidote), which has been frequently reported from secondary albite from granitic rocks worldwide, are absent in volcanic from Głuszyca.
3. AFT ages of the volcanic rocks range from 182 to 161 Ma, and hence span Early-Jurassic–Late-Jurassic time. These ages are significantly younger relative to the early-Permian volcanism (ca. 290 Ma) responsible for the formation of Głuszyca trachyandesite laccolith, but older than the large-scale geological events in the Intra-Sudetic Basin, such as tectonic inversion and exhumation. Thus, this time period can reflect the timing of low-temperature alterations typical of spilitzation in the Intra-Sudetic Basin.
4. Crystallization temperatures of replacive (Mg-rich) chlorites were estimated as ranging between 106–170 °C according to chemical and semi-empirical geothermometers. A similar temperature range was obtained for non-replacive (vugs-filling) chlorite found in close special association with celadonite. These results indicate that spilitization-related fluids were able to reheat volcanic rocks above the Apatite Partial Annealing zone, i.e. 70–110 °C and reset the AFT system.
5. Trace element variations (i.e. REEs) in variably-spilitized volcanic rocks are negligible except for LILE elements, which chiefly include Sr (the depletion in strongly-altered samples) and Cs (the enrichment in strongly altered samples). Meanwhile, the enrichment in LILE, Th, and LREE relative to Nb and Ta, coupled with humped-shaped mantle-normalized trace element patterns and positive Zr-Hf anomaly indicate metasomatism in the magma source (via subduction-related fluids), combined with the pronounced crustal contamination. Additionally, crustal contamination can be supported by the presence of interstitial quartz and yellow-luminescent (REE^3+^ and Mn^2+^ activated) outer domains of fluorapatite.

## Methods

The samples of volcanic rocks have been investigated using microscopic observations (optical microscopy, cathodoluminescence microscopy OM-CL and spectroscopy, scanning electron microscopy SEM), micro-chemical (electron microprobe measurements EMPA), and apatite fission-track dating (AFT). Total amount of ca. 20 rock samples have been initially collected from various parts of Głuszyca trachyandesite exposure, i.e.; lower (Loc. 1 in Fig. 2A), middle (Loc. 2 in Fig. 2A), and upper (Loc. 3 in Fig. 2A).

### Optical microscopy and cathodoluminescence (OM-CL)

Thin sections of the samples were examined using Olympus BX 51 polarizing microscope with a magnification ranging from 40 × to 400 × . The observations were conducted using transmitted (during micro-textural observations and description of main rock-forming components) and reflected light modes (for preliminary identification of opaque and ore-related minerals). The photomicrographs were acquired using an Olympus DP12 digital camera equipped with the Analysis software. The cathodoluminescence observations (OM-CL) were conducted at the Polish Geological Institute—National Research Institute in Warsaw. The CL observations were conducted on polished thin sections using a Cambridge Image Technology CCL 8200 MK3 device (cold cathode) coupled with a Nikon Optiphot 2 polarizing microscope. The CL photomicrographs have been taken using a digital Canon EOS 600D camera. Cathodoluminescence spectra were obtained by LEO 1430 scanning electron microscope coupled with a CL-image system (ASK-CL VIS View) and CL spectrometer (ASK SEM-CL). The system operated in a high-vacuum mode, at 20 kV accelerating voltage, and 50 μA current. The intensity of CL spectra was normalized to 100% in terms of the intensity units.

### Electron-microprobe analysis (EMPA) and chlorite geothermometry

Quantitative chemical analyses (electron microprobe analyses—EPMA) of fluorapatite, pyroxene, chlorite, primary plagioclase, and secondary albite were conducted at the Laboratory of Critical Elements of AGH University of Science and Technology—KGHM Polska Miedź S.A., using a JEOL Super Probe JXA-8230. Peak count-time of ca. 20 s and background time of 10 s were used during particular measurements. The system operated in a wavelength-dispersive (WDS) mode under high-vacuum conditions. Electron microprobe operating conditions, standards, and approximate detection limits are presented in Supplementary Data 1. The JEOL ZAF procedure was used for the matrix correction of the raw data. The temperature of chlorite formation was calculated using five independent approaches, i.e.:

1. T (°C) = –61.92 + 160.99·Al^IV^(O_14_)—^41^
2. T (°C) = 4833.946–2817.776·Si^IV^(O_14_) + 419.858·(Si^IV^)^2^—^40^
3. T (°C) = 319·[Al^IV^(O_28_) + 0.1· (Fe/Fe + Mg)] − 69—^44^
4. T (°C) = –106.2·[Al^IV^(O_28_)–0.88·(Fe/Fe + Mg−0.34)] + 17.5—^43^
5. T (°C) = 106.2·[Al^IV^(O_28_)–0.48·(Fe/Fe + Mg)−0.163] + 17.5—^42^

For semi-empirical estimation of formation temperatures, graphical geothermometer introduced by Bourdelle and Cathelineau^45^ has also been applied. Additionally, the chlorites containing ∑(Ca + Na + K) > 0.5 wt.% have been discarded from geothermometric calculations due to the possibility of contamination (i.e. remnants of pyroxenes or submicroscopic inclusions of other phases such as calcite) and/or the presence of interstratified chlorite-smectite phases. The application of thermodynamic approach^75^ was excluded due to high (> 3 apfu) Si content among particular chlorite species.

### Raman micro-spectroscopy (RS)

Raman spectra of pyroxene-group minerals were recorded using Thermo Scientific DXR Raman microscope equipped with 10 × , 50 × and 100 × magnification objectives. The system operated in a confocal mode and worked in a backscatter geometry. The measurement were conducted on polished thin section using a 532-nm laser with 10mW laser power and exposure time of 10 s. The laser focus diameter was 1–2 μm. The spectra were corrected for background by the sextic polynomial method using Omnic software. The identification of mineral phases was supported by CrystalSleuth (http://rruff.info/) and OMNIC software.

### Apatite fission-track dating (AFT)

Apatite fission-track thermochronology by the external detector method^76^ was carried out at the Institute of Geological Sciences, Polish Academy of Sciences in Krakow (Poland). The external detector method and the ζ age calibration approach were used to determine the fission-track ages^77,78^. Polished grain mounts were etched for 20 s in 5 N HNO_3_ at 21 °C. The standard glass CN5 was used as a dosimeter to monitor the neutron flux. Thin flakes of low-U muscovite were used as external detectors. Samples together with age standards (Fish Canyon, Durango, and Mount Dromedary apatites) and CN5 standard glass dosimeters were irradiated with thermal neutron nominal flux of 9 × 10^15^n/cm^2^ at the Oregon State University TRIGA reactor in the USA. After the irradiation, the muscovite external detectors were etched for about 45 min in 40% HF to reveal the induced tracks. The spontaneous and induced tracks were counted by optical microscopy at 1250 × magnification using a NIKON Eclipse E-600, equipped with motorised stage, digitising tablet and drawing tube controlled by program FTStage 4.04. Data analyses and age calculations based on a Zeta value for CN5 ζ_CN5_ of 348.18 ± 6.52 were calculated using program Trackkey 4.2 and further presented in the Tables 8 and 9, as well as Figs. 12 and 13. All quoted AFT ages are “central ages”^79^, and the spread of single grain ages was assessed using the dispersion of the central age and chi-square test^80^. Additional calculations were performed using Binomfit software^81^ to evaluate the reliability of obtained ages. Single-crystal ages have been further included in Supplementary Data 2 (Binomfit software results) and Supplementary Data 3 (comparison between Trackkey and Binomfit software, with error bars calculated at given coincidence interval). In all analysed samples about 20 apatite grains were selected for analyses. Only clean, defect- and inclusion-free grains were selected for track counting. The etch pit diameter (Dpar) was used to check annealing kinetics and the composition of apatites. At least four etch pits^82^ per single analysed grain have been measured. The crystals chosen for confined track measurements had a well-polished surface, parallel to the c-axis. For each sample, as many confined track lengths as possible were measured^83^.

### Whole rock major and trace element geochemistry

Seven samples have been selected for the whole-rock major- and trace-element analysis, which has been conducted at the Bureau Veritas Minerals Laboratories Ltd. in Vancouver Canada, using LF200 package. The material of 5 g per each sample was crushed using Abih mortar and pulverized using agate grinding mill. The material was then sieved to obtain ≥ 85% of fraction < 75 µm. The samples prepared in this manner were mixed with lithium tetraborate (Li_2_B_4_O_7_) flux. The crucibles were fused in a furnace. The cooled bead was dissolved in ACS grade nitric acid and analyzed by combined ICP-OES (Inductively Coupled Plasma—Optical Emission Spectrometry) and ICP-MS (Inductively Coupled Plasma—Mass Spectrometry) techniques. The major and trace elements were determined using Spectro Ciros Vision and ELAN 9000 devices, respectively. Loss on ignition (LOI) was measured by igniting a sample split followed by measuring of the weight loss.

## Supplementary Information

Supplementary Information 1.

Supplementary Information 2.

Supplementary Information 3.

## Data Availability

All data generated or analysed during this study are included in this published article (and its supplementary information files).
