# Supplementary material for: Origin and timing of spilitic alterations in volcanic rocks from Głuszyca Górna in the Intra-Sudetic Basin, Poland
Source: Sci Rep. 2022 Jul 11;12:11745. doi: 10.1038/s41598-022-15644-2 (PMC9273604; doi:10.1038/s41598-022-15644-2)
Supplement: Supplementary file 1 — Supplementary Information 1. [file 41598_2022_15644_MOESM1_ESM.docx]

| Pyroxene | | | | |  | Plagioclase/secondary albite | | | | | Fluorapatite | | | | |  | Chlorite | | | | |
| --- | --- | --- | --- | --- | --- | --- | --- | --- | --- | --- | --- | --- | --- | --- | --- | --- | --- | --- | --- | --- | --- |
| Acceleration voltage – 15 kV; Beam current – 10 nA beam size of 1-5 μm | | | | |  | Acceleration voltage – 15 kV; Beam current – 10 nA beam size of 3-5 μm | | | | | Acceleration voltage – 15 kV; Beam current – 10 nA beam size of 3-5 μm | | | | |  | Acceleration voltage – 15 kV; Beam current – 10 nA beam size of 5 μm | | | | |
| El. | Sig. | WDS cryst. | Std. | Det. lm. (ppm) |  | El. | Sig. | WDS cryst. | Std. | Det. lm. (ppm) | El. | Sig. | WDS cryst. | Std. | Det. lm.  (ppm) |  | El. | Sig. | WDS cryst. | Std. | Det. lm. (ppm) |
| Si | Kα | TAP | Albite | 400 |  | Si | Kα | TAP | Albite | 400 | Si | Kα | TAP | Albite | 400 |  | Si | Kα | TAP | Albite | 350 |
| Ti | Kα | PET | Rutile | 300 |  | Ti | Kα | PET | Rutile | 200 | Ca | Kα | PET | Diopside | 400 |  | Ti | Kα | PET | Rutile | 400 |
| Al | Kα | TAP | Albite | 500 |  | Al | Kα | TAP | Albite | 500 | Mn | Kα | LIF | Rhodonite | 900 |  | Al | Kα | TAP | Albite | 400 |
| Fe | Kα | LIF | Fayalite | 800 |  | Fe | Kα | LIF | Fayalite | 400 | Fe | Kα | LIF | Fayalite | 600 |  | Mn | Kα | LIF | Rhodonite | 600 |
| Mn | Kα | LIF | Rhodonite | 800 |  | Mg | Kα | TAP | Diopside | 300 | Mg | Kα | TAP | Diopside | 300 |  | Fe | Kα | LIF | Fayalite | 400 |
| Mg | Kα | TAP | Diopside | 300 |  | Ca | Kα | PET | Diopside | 150 | Na | Kα | TAP | Albite | 300 |  | Mg | Kα | TAP | Diopside | 250 |
| Ca | Kα | PET | Diopside | 300 |  | Na | Kα | TAP | Albite | 300 | La | Lα | LIF | LaPO_4_ | 900 |  | Ca | Kα | PET | Diopside | 150 |
| Na | Kα | TAP | Albite | 300 |  | K | Kα | PET | Sanidine | 200 | Ce | Lα | LIF | CePO_4_ | 700 |  | Na | Kα | TAP | Albite | 250 |
|  |  |  |  |  |  |  |  |  |  |  | Nd | Lα | LIF | NdPO_4_ | 800 |  | K | Kα | PET | Sanidine | 200 |
|  |  |  |  |  |  |  |  |  |  |  | Y | Kα | PET | YPO_4_ | 500 |  | F | Kα | LDE1 | Fluorite | 900 |
|  |  |  |  |  |  |  |  |  |  |  | Cl | Kα | PET | Tugtupite | 150 |  | Cl | Kα | PET | Tugtupite | 100 |
|  |  |  |  |  |  |  |  |  |  |  | F | Kα | LDE1 | Fluorite | 400 |  |  |  |  |  |  |

Electron microprobe conditions for particular phases that have been investigated in this study

Note: LIF - lithium fluoride, PET - pentaerythritol (PET), TAP - thallium acid pthalate, and LDE - artificial layered dispersive element; Detection limits, determined by JEOL software for 3σ confidence level, may vary in particular analytical points and hence only maximum values were presented
