# Supplementary material for: Origin and timing of spilitic alterations in volcanic rocks from Głuszyca Górna in the Intra-Sudetic Basin, Poland
Source: Sci Rep. 2022 Jul 11;12:11745. doi: 10.1038/s41598-022-15644-2 (PMC9273604; doi:10.1038/s41598-022-15644-2)

Datafile: D:\GL_01A.FTA
Title: GL1, trachyandesite;CN-5 glass for monitor

NEW PARAMETERS - ZETA METHOD

EFFECTIVE TRACK DENSITY FOR FLUENCE MONITOR (tracks/cm^2): 1.19E+06
RELATIVE ERROR (%): 1.68
EFFECTIVE URANIUM CONTENT OF MONITOR (ppm): 11.00
ZETA FACTOR AND STANDARD ERROR (yr cm^2): 348.18 6.52
SIZE OF COUNTER SQUARE (cm^2): 6.01E-07

GRAIN AGES IN ORIGINAL ORDER

Grain no.	RhoS (cm^-2)	(Ns)	RhoI (cm^-2)	(Ni)	Squares	U+/-2s		Grain Age (Ma) Age	--95% CI--	
1	4.16E+05	(4)	4.16E+05	(4)	16	4	4	203.1	38.3	1022.6
2	2.78E+05	(2)	2.78E+05	(2)	12	3	3	203.1	14.9	2358.1
3	5.55E+05	(4)	5.55E+05	(4)	12	5	5	203.1	38.3	1022.6
4	1.67E+05	(3)	3.33E+05	(6)	30	3	2	104.9	16.7	466.0
5	2.78E+05	(2)	2.78E+05	(2)	12	3	3	203.1	14.9	2358.1
6	4.76E+05	(4)	3.57E+05	(3)	14	3	4	266.1	46.4	1648.6
7	2.78E+05	(2)	2.78E+05	(2)	12	3	3	203.1	14.9	2358.1
8	2.78E+05	(3)	1.85E+05	(2)	18	2	2	295.4	35.3	2928.1
9	3.33E+05	(6)	3.33E+05	(6)	30	3	2	203.1	54.9	729.3
10	1.04E+05	(1)	2.08E+05	(2)	16	2	2	108.9	1.7	1728.3
11	4.16E+05	(3)	6.94E+05	(5)	12	6	5	125.1	19.2	607.1
POOLED	3.08E+05	(34)	3.44E+05	(38)	184	3	1	182.1	111.6	294.7

CHI^2 PROBABILITY (%): 99.7
>>> Beware: possible upward bias in Chi^2 probability due to low counts <<<

POOLED AGE W/ 68% CONF. INTERVAL(Ma): 182.1, 140.4 -- 235.7 (-41.7 +53.6)
95% CONF. INTERVAL(Ma): 111.6 -- 294.7 (-70.5 +112.6)

CENTRAL AGE W/ 68% CONF. INTERVAL(Ma): 182.0, 144.0 -- 229.9 (-38.0 +47.9)
95% CONF. INTERVAL(Ma): 114.9 -- 287.4 (-67.1 +105.5)
AGE DISPERSION (%): 0.1

FIT OPTION: Best-fit peaks using the binomial model of Galbraith and Green

INITIAL GUESS FOR MODEL PARAMETERS (number of peaks to fit = 2)

Peak #.	Peak Age	Theta	Fraction(%)	Count
1.	203.30	0.500	27.8	3.06
2.	110.10	0.350	16.2	1.78

Total range for grain ages: 110.1 to 282.5 Ma
Number of active grains (Num. used for fit): 11
Number of removed grains: 0
Degrees of freedom for fit: 8
Average of the SE(Z)'s for the grains: 0.78
Estimated width of peaks in PD plot in Z units: 0.91

PARAMETERS FOR BEST-FIT PEAKS

* *Standard error for peak age includes group error*
* *Peak width is for PD plot assuming a kernel factor = 0.60*

#.	Peak Age(Ma)	68%CI	95%CI	W(Z)	Frac(%)	SE,%	Count
1.	182.0	-44.1 ...+58.0	-76.5 ...+130.6	0.91	67.3	3341682.0	7.4
2.	182.0	-17.2 ...+19.0	-32.2 ...+39.0	0.91	32.7	3341682.0	3.6

Log-likelihood for best fit: -13.682
Chi-squared value for best fit: 1.960
Reduced chi-squared value: 0.245
Probability for F test: 100%
Condition number for COVAR matrix: 12729040000.00
Number of iterations: 7

Datafile: D:\GL_01A.FTA
Title: GL1, trachyandesite;CN-5 glass for monitor

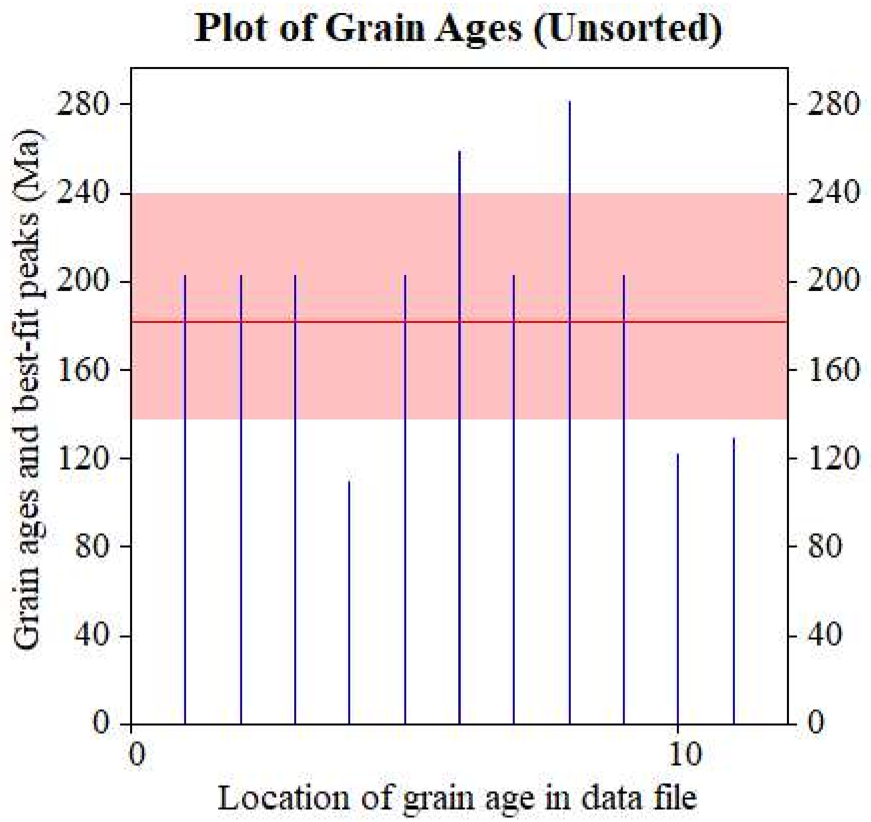

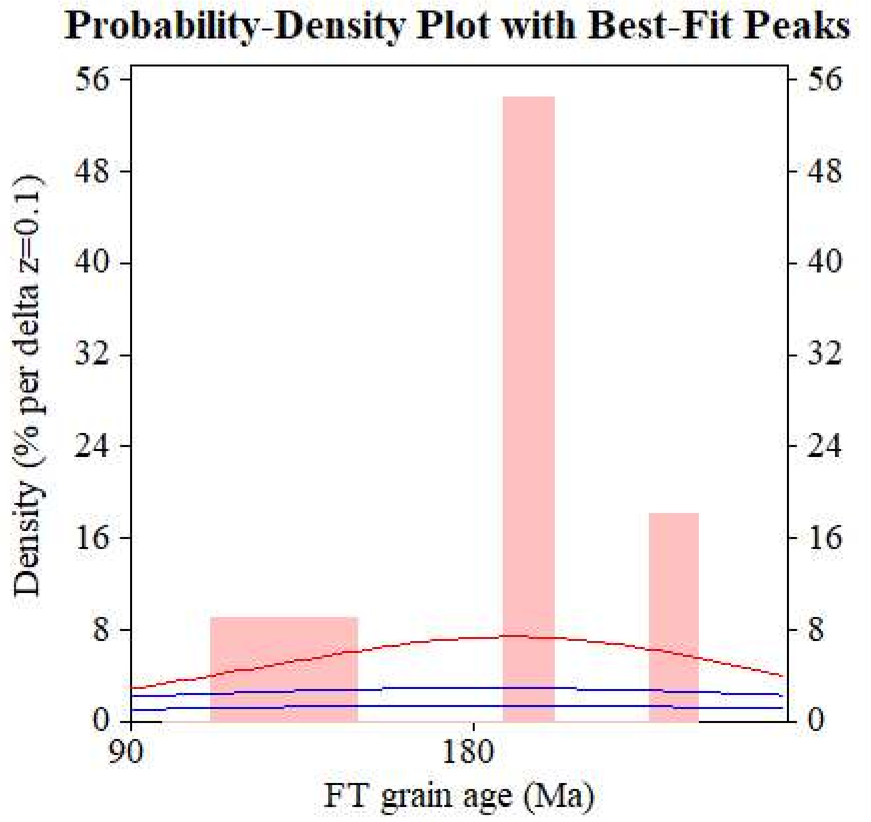

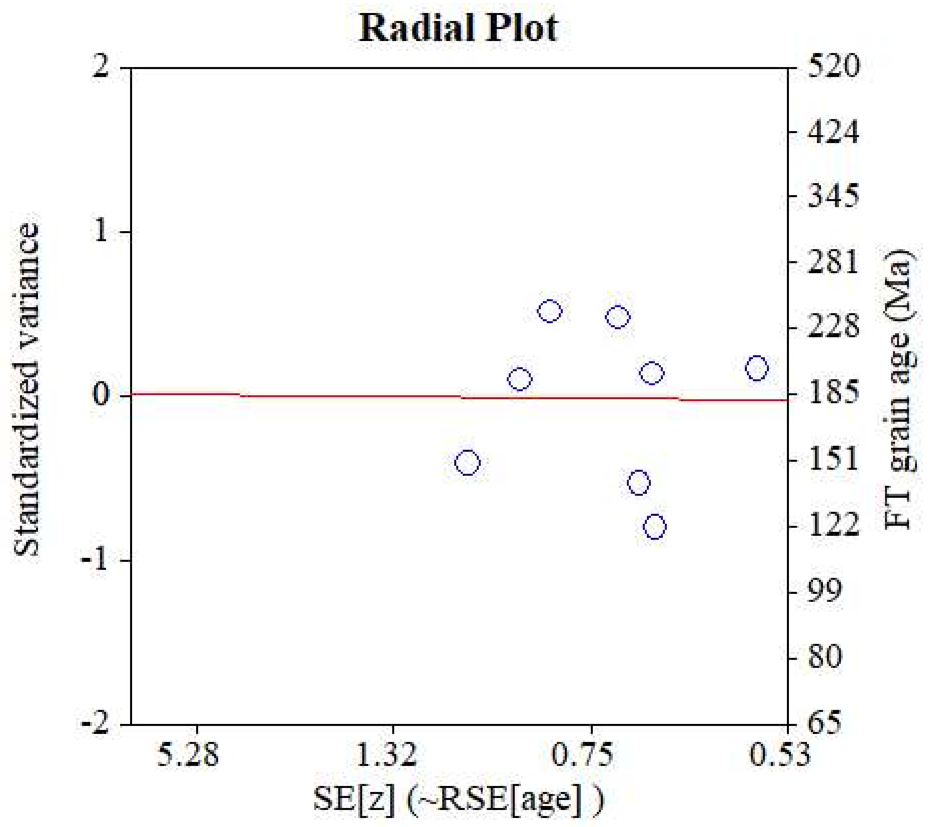
Radial Plot
Standardized variance
2
1
0
-1
-2
5.28
1.32
0.75
0.53
SE[z] (~RSE[age])
FT grain age (Ma)
520
424
345
281
228
185
151
122
99
80
65

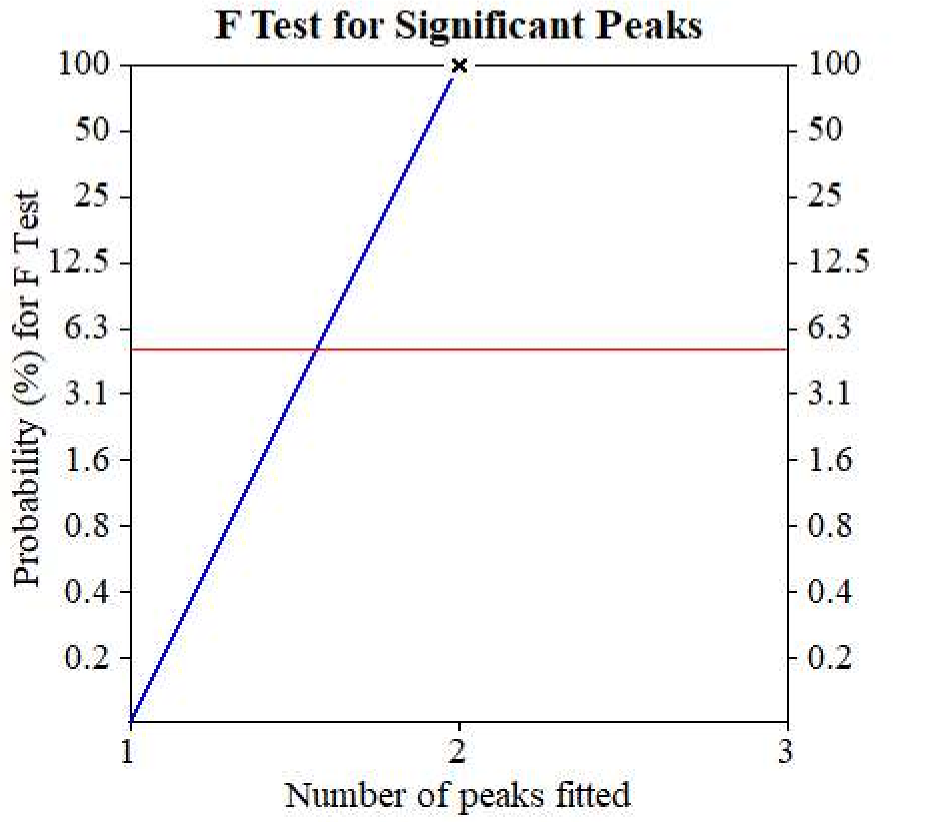
F Test for Significant Peaks
Probability (%) for F Test
100
50
25
12.5
6.3
3.1
1.6
0.8
0.4
0.2
1
2
3
Number of peaks fitted

Datafile: D:\GL_2.ftz
Title: GL2, trachybazalt;CN-5 glass for monitor

NEW PARAMETERS - ZETA METHOD

EFFECTIVE TRACK DENSITY FOR FLUENCE MONITOR (tracks/cm^2): 1.20E+06
RELATIVE ERROR (%): 1.68
EFFECTIVE URANIUM CONTENT OF MONITOR (ppm): 11.00
ZETA FACTOR AND STANDARD ERROR (yr cm^2): 348.18 6.52
SIZE OF COUNTER SQUARE (cm^2): 6.01E-07

GRAIN AGES IN ORIGINAL ORDER

Grain no.	RhoS (cm^-2)	(Ns)	RhoI (cm^-2)	(Ni)	Squares	U+/-2s		Grain Age (Ma)	--95% CI--	
1	4.16E+05	3	5.55E+05	4	12	5	5	156.2	22.8	863.1
2	3.33E+05	3	4.44E+05	4	15	4	4	156.2	22.8	863.1
3	2.50E+05	3	2.50E+05	3	20	2	2	204.9	27.8	1393.2
4	3.33E+05	3	3.33E+05	3	15	3	3	204.9	27.8	1393.2
5	1.59E+05	2	2.38E+05	3	21	2	2	140.6	11.6	1110.8
6	1.67E+05	3	1.67E+05	3	30	2	2	204.9	27.8	1393.2
7	1.39E+05	1	1.39E+05	1	12	1	2	204.9	2.7	8141.8
8	4.16E+05	3	4.16E+05	3	12	4	4	204.9	27.8	1393.2
9	2.08E+05	2	4.16E+05	4	16	4	4	107.0	9.4	688.7
10	2.38E+05	3	3.17E+05	4	21	3	3	156.2	22.8	863.1
11	1.11E+05	1	3.33E+05	3	15	3	3	75.2	1.3	811.5
12	2.22E+05	2	3.33E+05	3	15	3	3	140.6	11.6	1110.8
13	3.33E+05	3	3.33E+05	3	15	3	3	204.9	27.8	1393.2
14	1.39E+05	1	1.39E+05	1	12	1	2	204.9	2.7	8141.8
15	5.95E+05	5	3.57E+05	3	14	3	4	331.5	67.1	1919.3
16	9.25E+04	1	2.78E+05	3	18	3	3	75.2	1.3	811.5
17	3.12E+05	3	3.12E+05	3	16	3	3	204.9	27.8	1393.2
18	3.33E+05	3	4.44E+05	4	15	4	4	156.2	22.8	863.1
19	2.78E+05	2	2.78E+05	2	12	3	3	204.9	15.1	2376.4
POOLED	2.56E+05	47	3.10E+05	57	306	3	1	169.6	113.0	252.5

CHI^2 PROBABILITY (%):100.0
>>> Beware: possible upward bias in Chi^2 probability due to low counts <<<

POOLED AGE W/ 68% CONF. INTERVAL(Ma): 169.6, 136.9 -- 209.6 (-32.7 +40.0)
95% CONF. INTERVAL(Ma): 113.0 -- 252.5 (-56.6 +83.0)

CENTRAL AGE W/ 68% CONF. INTERVAL(Ma): 169.4, 139.2 -- 206.1 (-30.2 +36.6)
95% CONF. INTERVAL(Ma): 115.3 -- 248.5 (-54.2 +79.1)
AGE DISPERSION (%): 0.0

FIT OPTION: Best-fit peaks using the binomial model of Galbraith and Green

INITIAL GUESS FOR MODEL PARAMETERS (number of peaks to fit = 2)

Peak #.	Peak Age	Theta	Fraction(%)	Count
1.	176.80	0.463	26.7	5.08
2.	110.10	0.348	18.2	3.45

Total range for grain ages: 88.6 to 319.2 Ma
Number of active grains (Num. used for fit): 19
Number of removed grains: 0
Degrees of freedom for fit: 16
Average of the SE(Z)'s for the grains: 0.84
Estimated width of peaks in PD plot in Z units: 0.98

PARAMETERS FOR BEST-FIT PEAKS

* *Standard error for peak age includes group error*
* *Peak width is for PD plot assuming a kernel factor = 0.60*

#.	Peak Age(Ma)	68%CI	95%CI	W(Z)	Frac(%)	SE,%	Count
1.	169.4	-34.9 ...+43.8	-61.6 ...+96.2	1.00	62.3	1347294.0	11.8
2.	169.4	-19.4 ...+21.9	-36.0 ...+45.5	1.00	37.7	1347294.0	7.2

Log-likelihood for best fit: -22.121
Chi-squared value for best fit: 3.206
Reduced chi-squared value: 0.200
Probability for F test: 100%
Condition number for COVAR matrix: 2753464000.00
Number of iterations: 6

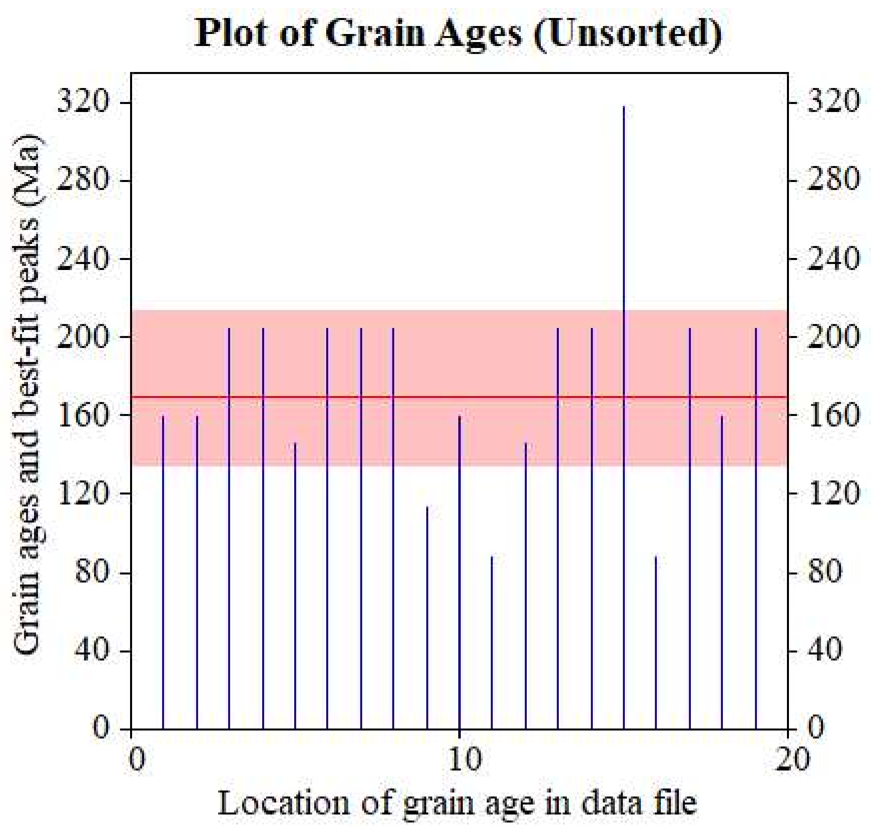
Plot of Grain Ages (Unsorted)
Grain ages and best-fit peaks (Ma)
320
280
240
200
160
120
80
40
0
0
10
20
Location of grain age in data file

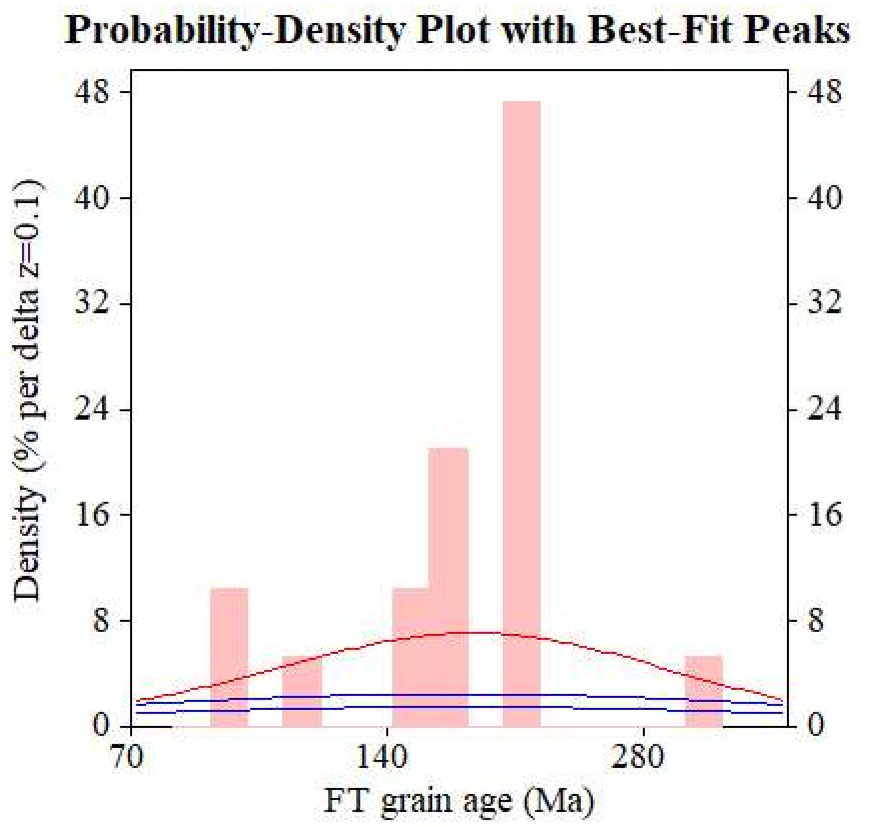
Probability-Density Plot with Best-Fit Peaks
Density (% per delta z=0.1)
48
40
32
24
16
8
0
70
140
280
FT grain age (Ma)

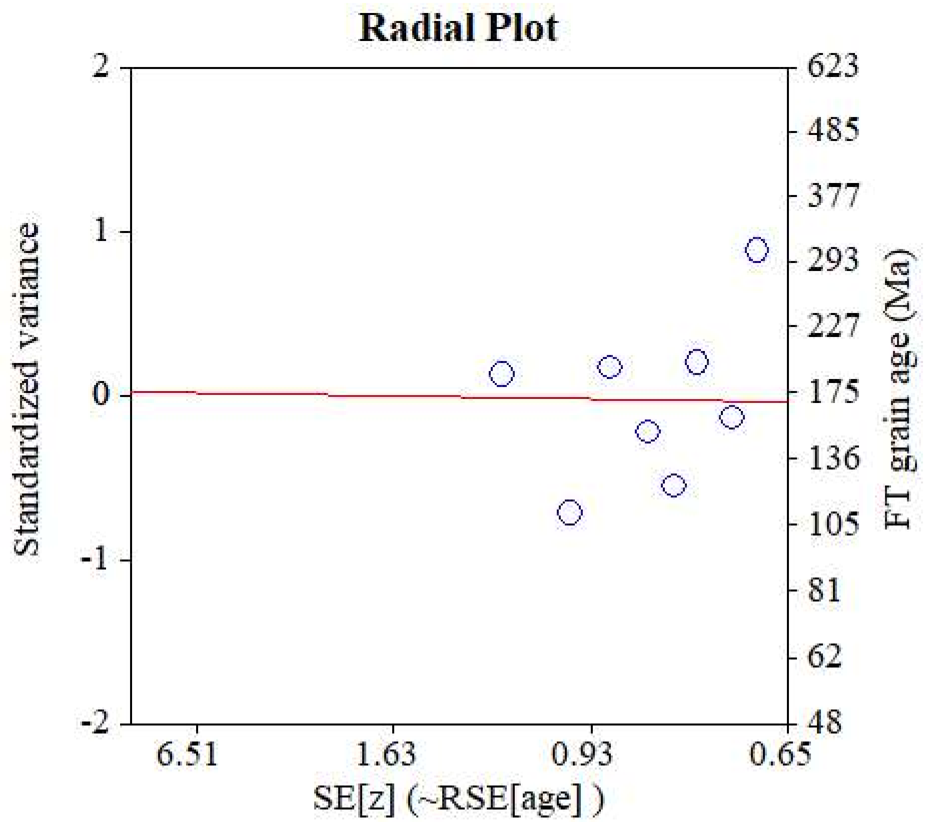
Radial Plot
Standardized variance
2
1
0
-1
-2
623
485
377
293
227
175
136
105
81
62
48
FT grain age (Ma)
6.51
1.63
0.93
0.65
SE[z] (~RSE[age])

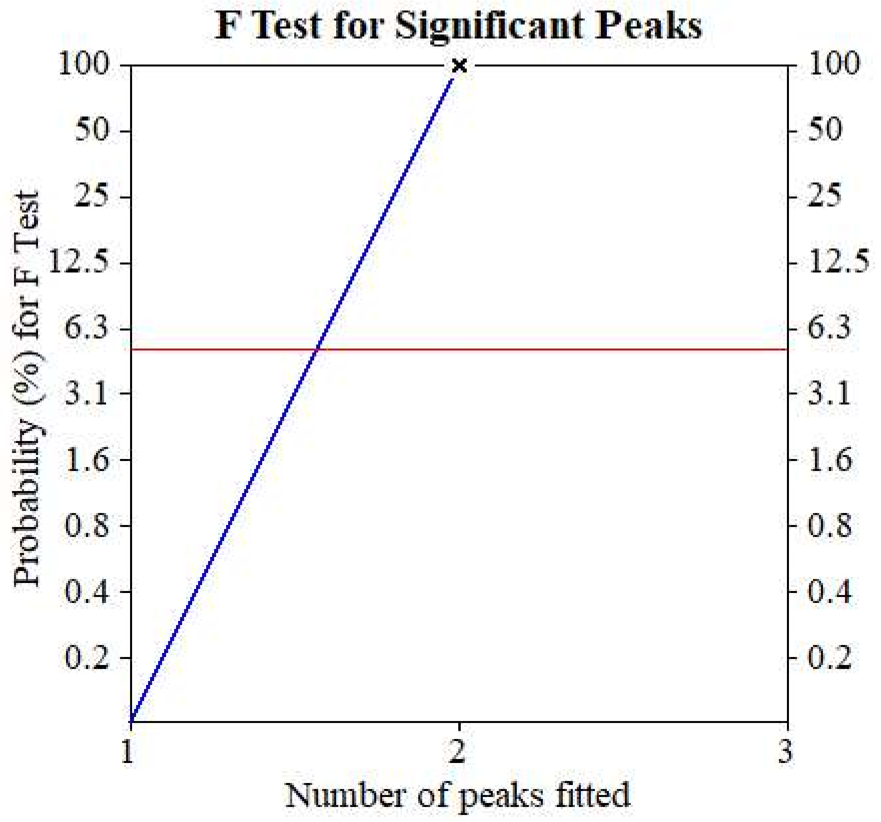
F Test for Significant Peaks
Probability (%) for F Test
100
50
25
12.5
6.3
3.1
1.6
0.8
0.4
0.2
1
2
3
Number of peaks fitted

Datafile: D:\GL_3.FTA
Title: GL3, trachybazalt;CN-5 glass for monitor

NEW PARAMETERS - ZETA METHOD

EFFECTIVE TRACK DENSITY FOR FLUENCE MONITOR (tracks/cm^2): 1.19E+06
RELATIVE ERROR (%): 1.68
EFFECTIVE URANIUM CONTENT OF MONITOR (ppm): 11.00
ZETA FACTOR AND STANDARD ERROR (yr cm^2): 348.18 6.52
SIZE OF COUNTER SQUARE (cm^2): 6.01E-07

GRAIN AGES IN ORIGINAL ORDER

Grain no.	RhoS (cm^-2)	(Ns)	RhoI (cm^-2)	(Ni)	Squares	U+/-2s		Grain Age (Ma)	--95% CI--	
1	3.33E+05	3	3.33E+05	3	15	3	3	204.3	27.7	1389.0
2	1.11E+05	1	2.22E+05	2	15	2	3	109.5	1.8	1737.2
3	2.38E+05	2	2.38E+05	2	14	2	3	204.3	15.0	2369.7
4	2.78E+05	2	2.78E+05	2	12	3	3	204.3	15.0	2369.7
5	2.78E+05	2	2.78E+05	2	12	3	3	204.3	15.0	2369.7
6	2.22E+05	2	3.33E+05	3	15	3	3	140.1	11.5	1107.4
7	2.78E+05	3	4.63E+05	5	18	4	4	125.8	19.3	610.5
8	2.78E+05	2	2.78E+05	2	12	3	3	204.3	15.0	2369.7
9	3.70E+05	4	4.63E+05	5	18	4	4	165.2	32.8	728.9
10	2.78E+05	2	4.16E+05	3	12	4	4	140.1	11.5	1107.4
11	1.85E+05	2	1.85E+05	2	18	2	2	204.3	15.0	2369.7
12	2.22E+05	2	2.22E+05	2	15	2	3	204.3	15.0	2369.7
13	1.11E+05	1	2.22E+05	2	15	2	3	109.5	1.8	1737.2
14	2.22E+05	2	2.22E+05	2	15	2	3	204.3	15.0	2369.7
15	1.39E+05	1	1.39E+05	1	12	1	2	204.3	2.6	8126.3
16	4.16E+05	3	2.78E+05	2	12	3	3	297.1	35.6	2941.9
17	3.17E+05	4	4.76E+05	6	21	4	3	138.6	28.6	558.8
18	1.59E+05	2	2.38E+05	3	21	2	2	140.1	11.5	1107.4
19	1.39E+05	1	5.55E+05	4	12	5	5	57.0	1.1	504.3
20	3.33E+05	3	3.33E+05	3	15	3	3	204.3	27.7	1389.0
POOLED	2.45E+05	44	3.12E+05	56	299	3	1	161.2	106.3	242.2

CHI^2 PROBABILITY (%):100.0
>>> Beware: possible upward bias in Chi^2 probability due to low counts <<<

POOLED AGE W/ 68% CONF. INTERVAL(Ma): 161.2, 129.4 -- 200.2 (-31.8 +39.1)
95% CONF. INTERVAL(Ma): 106.3 -- 242.2 (-54.9 +81.0)

CENTRAL AGE W/ 68% CONF. INTERVAL(Ma): 161.0, 131.7 -- 196.7 (-29.3 +35.7)
95% CONF. INTERVAL(Ma): 108.6 -- 238.3 (-52.4 +77.3)
AGE DISPERSION (%): 0.1

FIT OPTION: Best-fit peaks using the binomial model of Galbraith and Green

INITIAL GUESS FOR MODEL PARAMETERS (number of peaks to fit = 2)

Peak #.	Peak Age	Theta	Fraction(%)	Count
1.	166.60	0.449	26.2	5.24
2.	110.10	0.349	18.7	3.73

Total range for grain ages: 68.8 to 284.2 Ma
Number of active grains (Num. used for fit): 20
Number of removed grains: 0
Degrees of freedom for fit: 17
Average of the SE(Z)'s for the grains: 0.87
Estimated width of peaks in PD plot in Z units: 1.01

PARAMETERS FOR BEST-FIT PEAKS

* *Standard error for peak age includes group error*
* *Peak width is for PD plot assuming a kernel factor = 0.60*

#.	Peak Age(Ma)	68%CI	95%CI	W(Z)	Frac(%)	SE,%	Count
1.	161.0	-34.2 ...+43.2	-60.2 ...+95.4	1.05	60.4	5566770.0	12.1
2.	161.0	-19.6 ...+22.2	-36.1 ...+46.4	1.05	39.6	5566770.0	7.9

Log-likelihood for best fit: -22.140
Chi-squared value for best fit: 2.879
Reduced chi-squared value: 0.169
Probability for F test: 100%
Condition number for COVAR matrix: 43965840000.00
Number of iterations: 6

Datafile: D:\GL_3.FTA
Title: GL3, trachybazalt;CN-5 glass for monitor

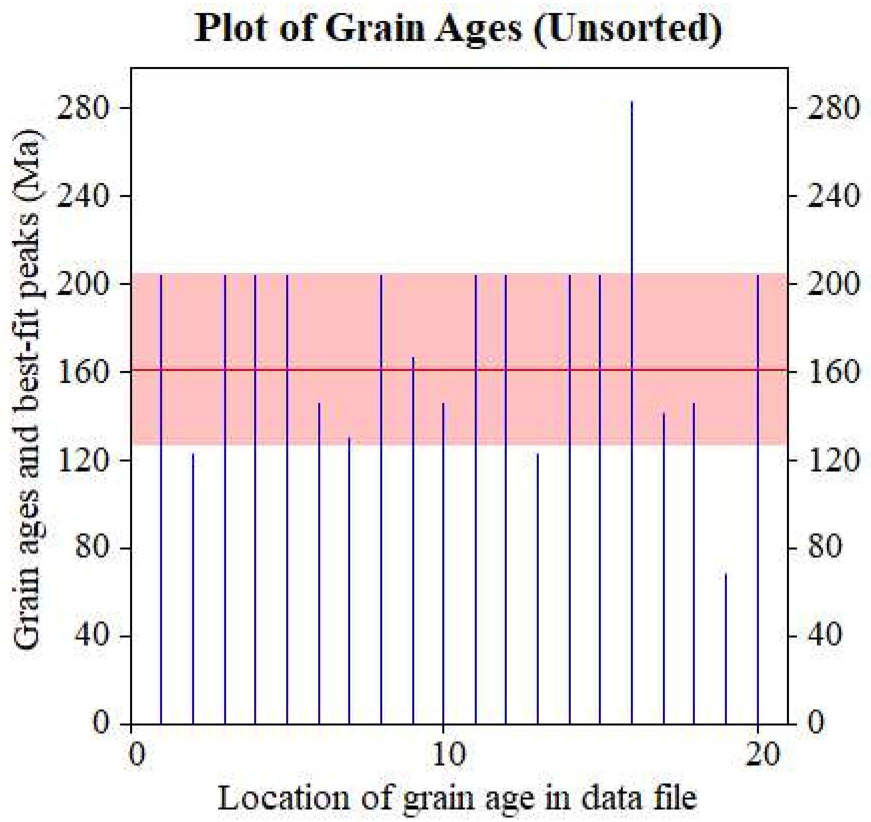

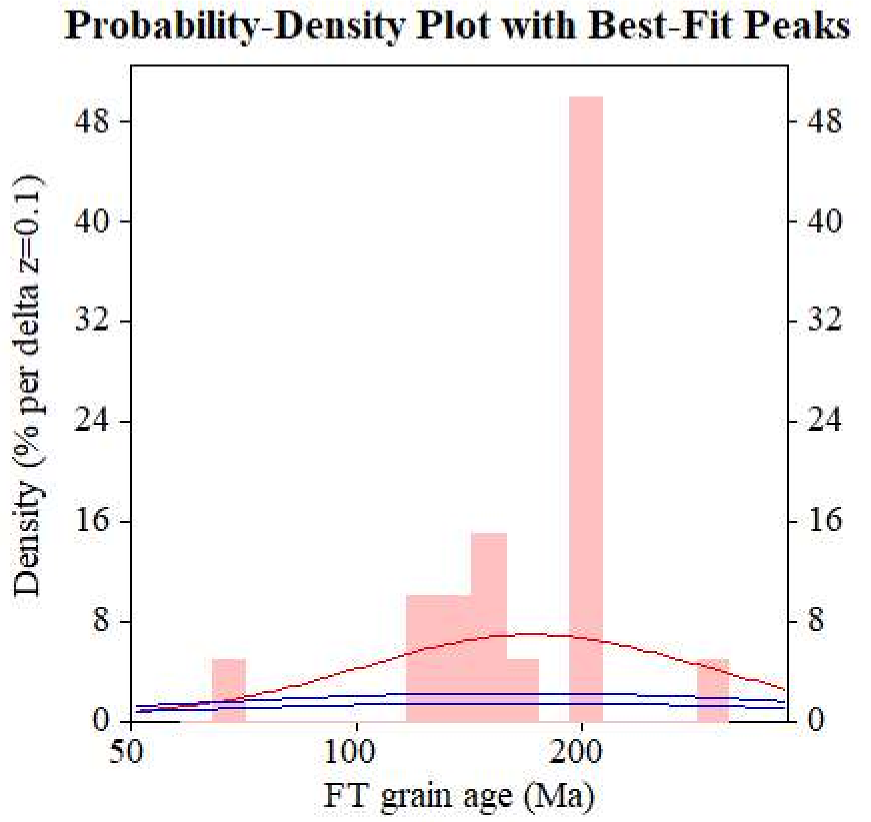

Datafile: D:\GL_3.FTA
Title: GL3, trachybazalt;CN-5 glass for monitor

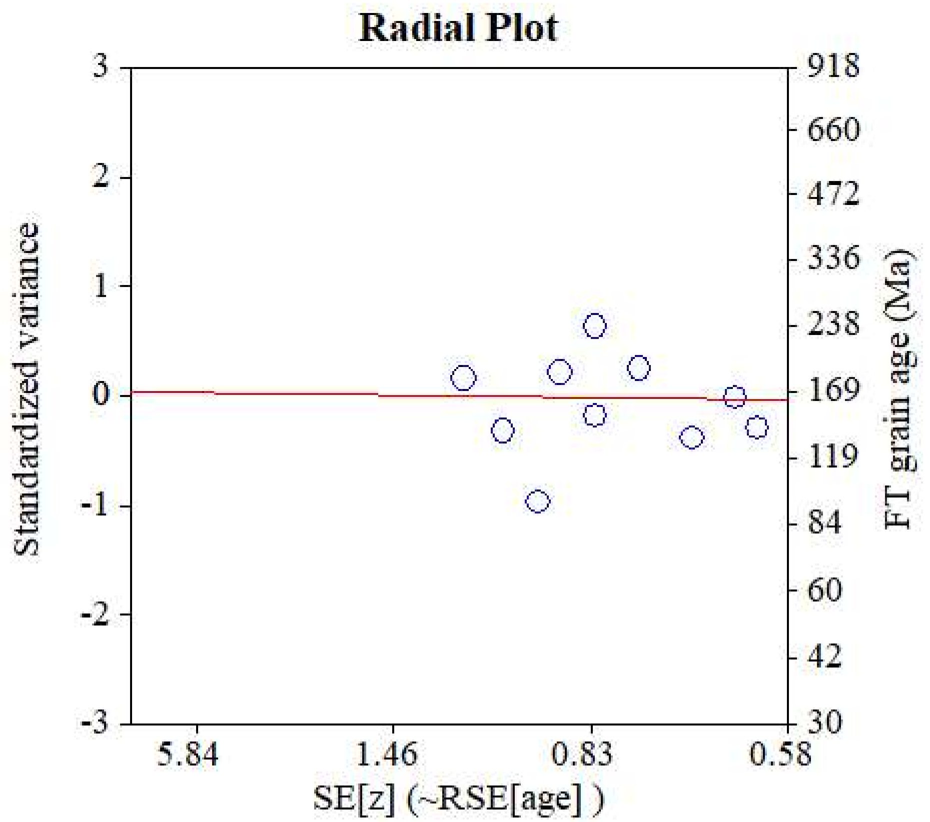

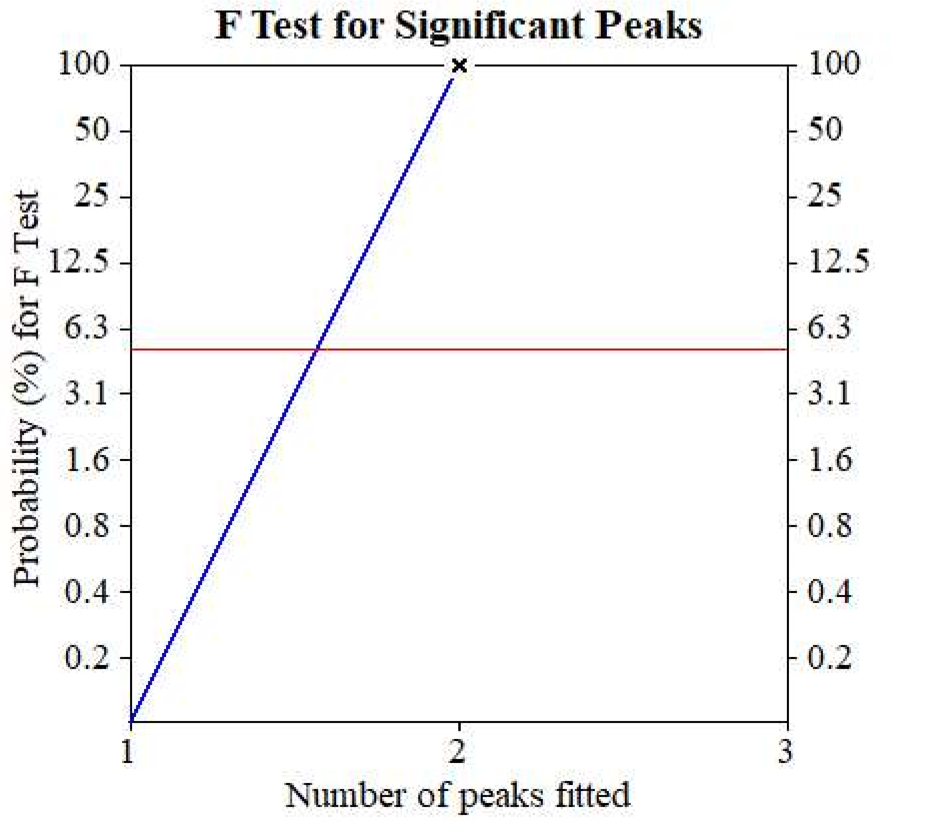

Supplement: Supplementary file 2 — Supplementary Information 2. [file 41598_2022_15644_MOESM2_ESM.pdf]
